# Recognition of extended linear and cyclised polyketide mimics by a type II acyl carrier protein†
†Electronic supplementary information (ESI) available: Detailed experimental procedures and characterisation data for all new compounds, additional spectra and structural statistics for derivatised ACP three-dimensional structures. See DOI: 10.1039/c5sc03864b



**DOI:** 10.1039/c5sc03864b

**Published:** 2015-12-10

**Authors:** Xu Dong, Christopher D. Bailey, Christopher Williams, John Crosby, Thomas J. Simpson, Christine L. Willis, Matthew P. Crump

**Affiliations:** a School of Chemistry , University of Bristol , Cantock's Close , Bristol , BS8 1TS , UK . Email: matt.crump@bristol.ac.uk ; Email: chris.willis@bristol.ac.uk

## Abstract

Polyketides are secondary metabolites which display both valuable pharmaceutical and agrochemical properties. Biosynthesis is performed by polyketide synthases (PKSs), and the acyl carrier protein (ACP), a small acidic protein, that transports the growing polyketide chain and is essential for activity. Here we report the synthesis of two aromatic probes and a linear octaketide mimic that have been tethered to actinorhodin ACP. These experiments were aimed at probing the ACP's capacity to sequester a non-polar *versus* a phenolic aromatic ring (that more closely mimics a polyketide intermediate) as well as investigations with extended polyketide chain surrogates. The binding of these mimics has been assessed using high-resolution solution NMR studies and high-resolution structure determination. These results reveal that surprisingly a PKS ACP is able to bind and sequester a bulky non-polar substrate containing an aromatic ring in a fatty acid type binding mode, but the introduction of even a small degree of polarity favours a markedly different association at a surface site that is distinct from that employed by fatty acid ACPs.

## Abstract 2

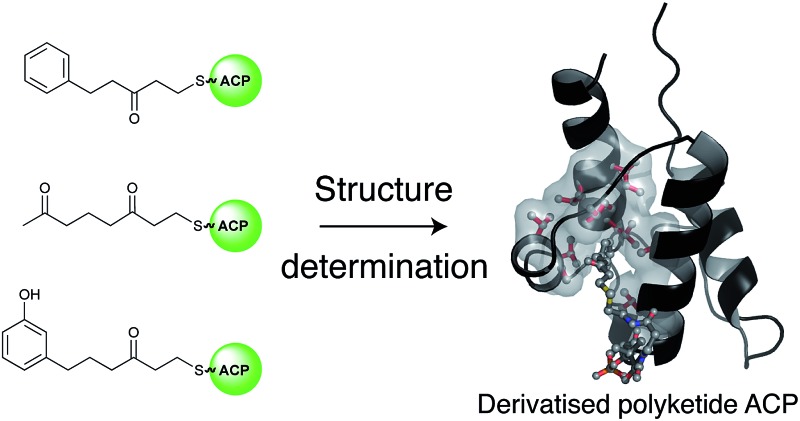
Extended linear and cyclised polyketide mimics were synthesized and high-resolution solution NMR structures were used to probe the interactions of the actinorhodin polyketide ACP with these surrogates.

## Introduction

**Fig. 1:**
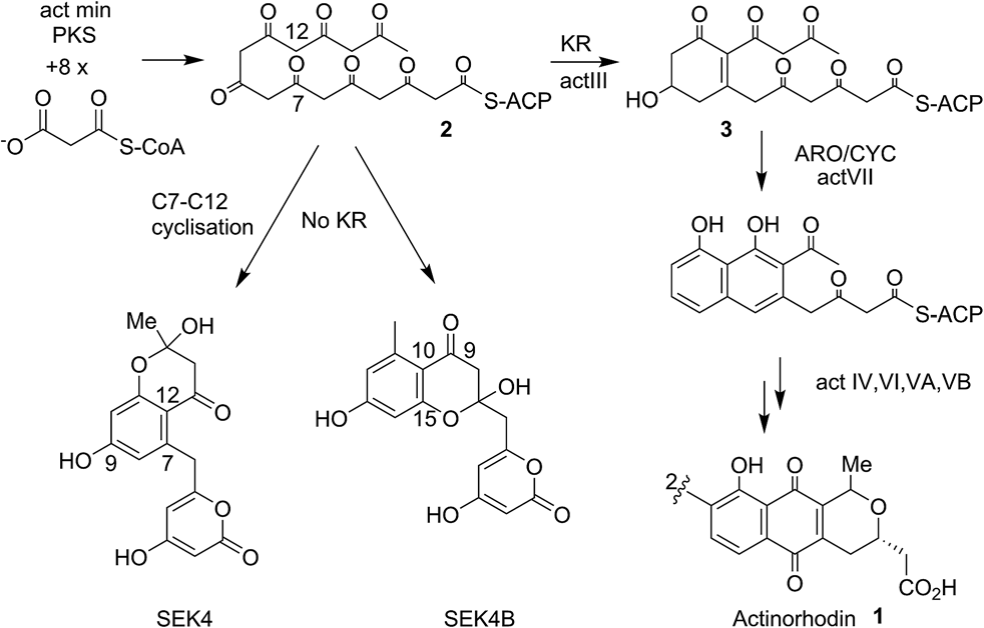
Proposed biosynthetic pathway to actinorhodin **1**. The polyketone **2** is attached to holo-ACP during the elongation of the polyketide backbone to the full sixteen carbon chain-length. Both studies *in vivo* and *in vitro* showed the proteins encoded by *actI* (KS/CLF and ACP), the so-called minimal PKS catalyses the generation of the full-length polyketide chain. The minimal PKS also partially controlled the C7–C12 ring closure to yield SEK4 as well as a mis-cyclised product formed from the C10–C15 ring closure (SEK4b).^6^ Ketide units are depicted in the keto-forms although these are likely to be a mixture of keto–enol tautomers.

Actinorhodin **1** (Fig. 1) produced by the archetype type II polyketide synthase from *Streptomyces coelicolor*, is an aromatic polyketide antibiotic that has served as a paradigm for the genetic, mechanistic and structural elucidation of aromatic polyketide biosynthesis.^1–4^ It is produced by a type II polyketide synthase, derived from a cluster of genes (23 in total) coding for each of the biosynthetic, self-resistance and export proteins associated with the actinorhodin pathway. Both studies *in vivo* and *in vitro* have shown that proteins encoded by *actI* (KS/CLF and ACP) form a minimal PKS^5^ that catalyses the generation of the full length polyketide chain 1 (Fig. 1). Successive reductive (*actIII*, KR), cyclisation/aromatisation (*actVII*, ARO/CYC) and further tailoring steps then complete the biosynthesis. Whereas these latter tailoring steps follow a simpler enzyme–substrate model, the initial chain elongation steps involve precisely seven iterative decarboxylative condensations of seven malonate units with a malonate derived acetate unit primer to give the desired chain length.

Studies of type II FAS ACPs with fully reduced^7,8^ or partially reduced fatty acids^9^ have revealed deep sequestration of the intermediate by the ACP and concomitant structural changes in the protein that may assist in modulating protein–protein interactions. Conversely the rat FAS type I ACP bearing a fully reduced fatty acid chain showed no interaction of the protein with the fatty acid side-chain by NMR as evidenced by no observable NOEs or chemical shift perturbations in ^1^H–^15^N HSQC spectra.^10^ This has provided a useful yardstick to define the opposite ends of these two extremes. Very little is actually known about the mechanism by which a linear polyketide is successfully stabilised and transferred from the KS/CLF to the next enzyme in the sequence, *actIII* KR. Primarily this is because the ACP-bound labile intermediates are not isolatable and we lack suitable stable chemical mimics to serve as probes for these critical elongation steps. The ideal side chains to study ACP-intermediate interactions would be the true polyketide intermediates but the intrinsically high reactivity of the native or postulated intermediates makes them unsuitable for structural studies and they are synthetically intractable. We have previously probed the structural role of act PKS ACP in binding and protection of the nascent polyketide using solution state NMR and acylated ACPs derived from chemically synthesised coenzyme A (CoASH) derivatives.^11^ To generate more stable mimics we utilised polyketide analogues that employ strategically removed carbonyls but retain essential features such as flexibility of the intermediate and chain polarity. A terminal enone moiety is incorporated to allow Michael addition of the free sulfhydryl group of CoASH to generate a stable thioether group. The intermediate and the 4′-phosphopantetheine (4′-PP) portion of CoASH is then transferred to the target ACP using ACPS.^12^ Using this strategy we were able to provide evidence that short diketide and triketide mimics show a weak association with the act PKS ACP^11^ and associated conformational exchange within the protein. NMR studies with emodin, a putative mimic of actinorhodin, have also suggested association with the ACP.^13^

All previous studies of early pathway intermediate analogues have, however, focused on short linear polar or non-polar chains, which surprisingly are sequestered by a PKS ACP. They have not addressed whether the ACP plays any role in interacting with longer or monocyclic intermediates that more closely resemble the critical assembly junctures in type II polyketide biosynthesis. Many questions remain unanswered. (1) The ACP's true role in stabilising longer polyketide chains is unknown. (2) The timing of the first ring cyclisation is speculated to occur on the KR^14^ and does the ACP bind the resulting reduced cyclised product **3**? Finally (3) what is the nature of the interaction of the ACP with these intermediates and will the ‘right’ intermediate show full sequestration like a fatty acid or does the true picture lie somewhere between this and the completely unbound model observed in type I FAS systems?^15^

**Scheme 1:**
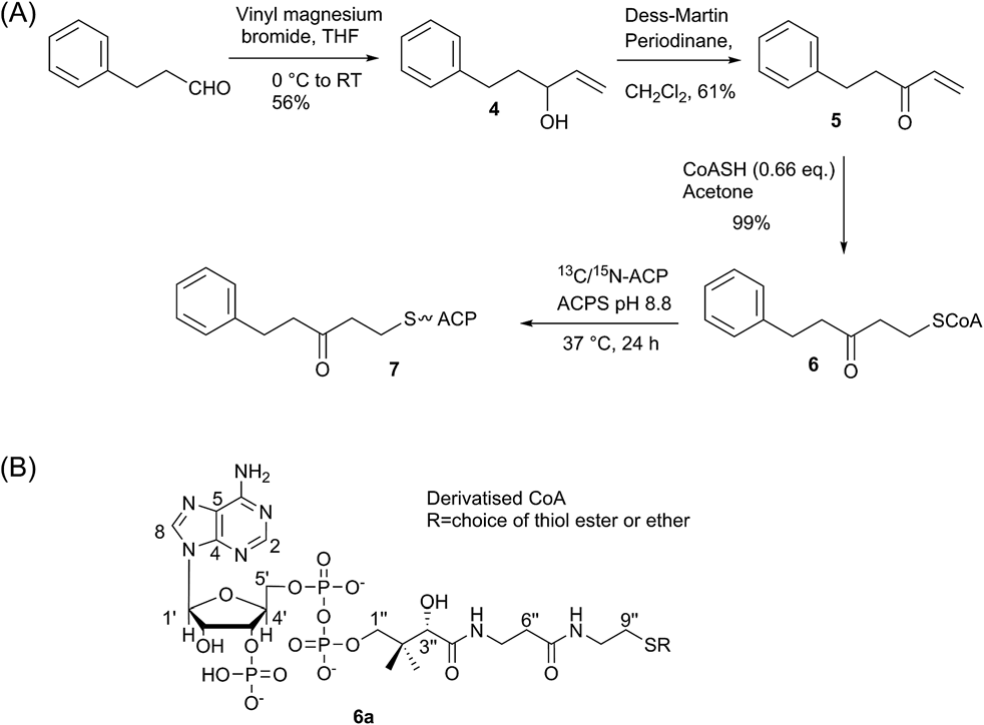
(A) Synthesis of 5-phenyl-3-oxo-pentyl ACP. (B) Generalised CoASH derivative.

**Scheme 2:**
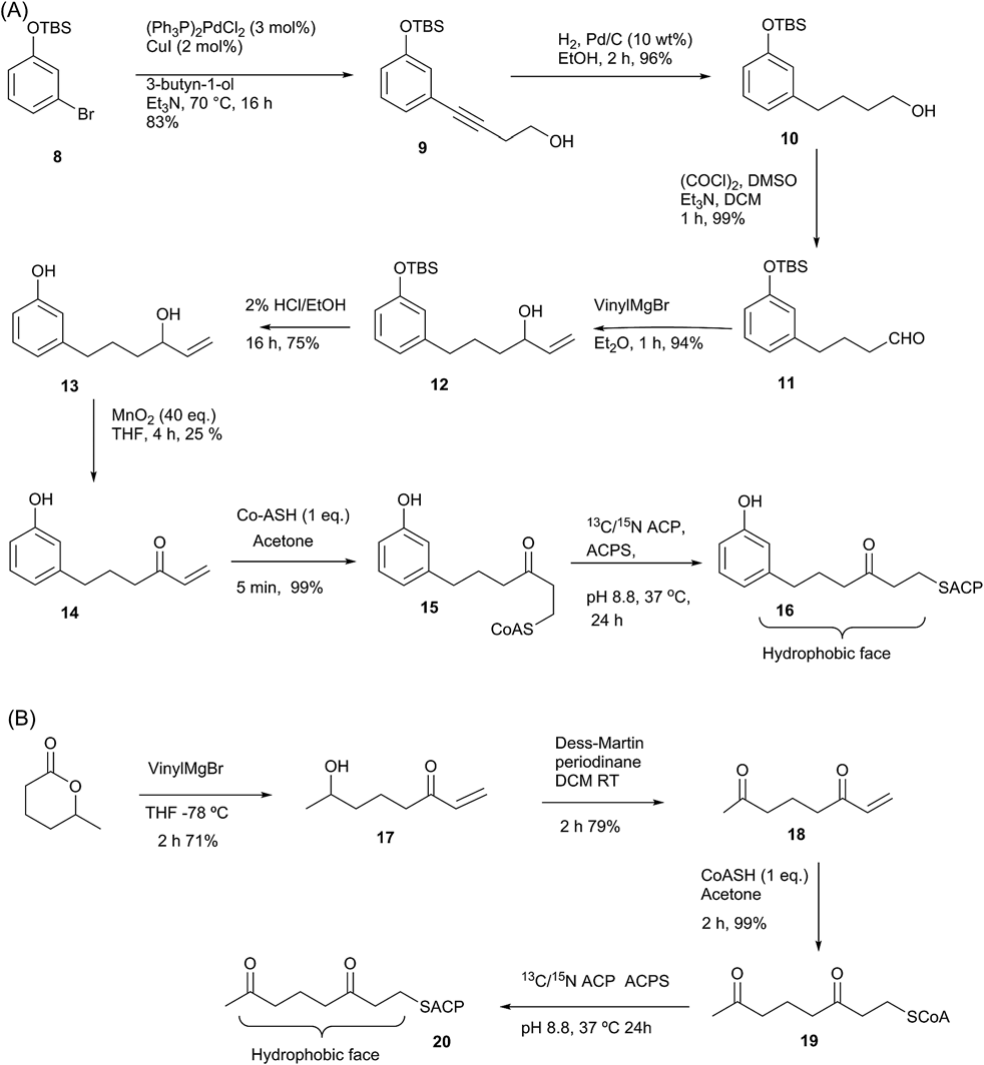
(A) Synthesis of phenol-derived enone **14** and derivatised ACP **16**. (B) Route to 3,7-dioxo-oct-1-ene **18** and derivatised ACP **20**.

Here we report the synthesis and structural characterisation of two aromatic derivatives and a linear octaketide surrogate covalently bound to the *S. coelicolor* actinorhodin (act) PKS ACP. The first, 5-phenyl-3-oxo-pentyl holo-ACP **7** (Scheme 1) contains an unmodified aromatic ring and a high resolution NMR structure shows it is buried within the hydrophobic core of the ACP. Two polar intermediates were then prepared, 6-(3-hydroxyphenyl)-3-oxo-hexyl holo ACP (Phenolic ACP) **16** (Scheme 2, below) containing a polar phenol group, was designed to more closely resemble an early stage polyketide intermediate and provide a comparison with the binding observed in 5-phenyl-3-oxo-pentyl holo-ACP. Similarly 3,7-dioxo-octyl ACP **20** (Scheme 2) would also provide a direct comparison with the tight binding and sequestration observed previously in the non-polar octyl ACP.^11^ In both cases we observe that the introduction of these moderate degrees of polarity can promote a rearrangement to an alternate binding mode where the derivative is associated with the surface of the ACP rather than within it.

## Results and discussion

### Synthesis and three-dimensional structure of 5-phenyl-3-oxo-pentyl ACP **7**

We began by synthesising 5-phenyl-3-oxo-pent-1-ene **5** in two steps *via* reaction of vinylmagnesium bromide with dihydrocinnamaldehyde followed by oxidation of allylic alcohol **4** (Scheme 1). Conjugate addition of CoASH to **5** gave **6** which was transferred by ACPS onto uniformly ^15^N-labelled apo act ACP to yield 5-phenyl-3-oxo-pentyl holo-ACP **7**. This ligand was designed to determine whether the ACP is capable of adjusting to accommodate a non-substituted aromatic ring albeit with reduced functionality compared to the postulated early actinorhodin intermediate (Fig. 1).

**Fig. 2:**
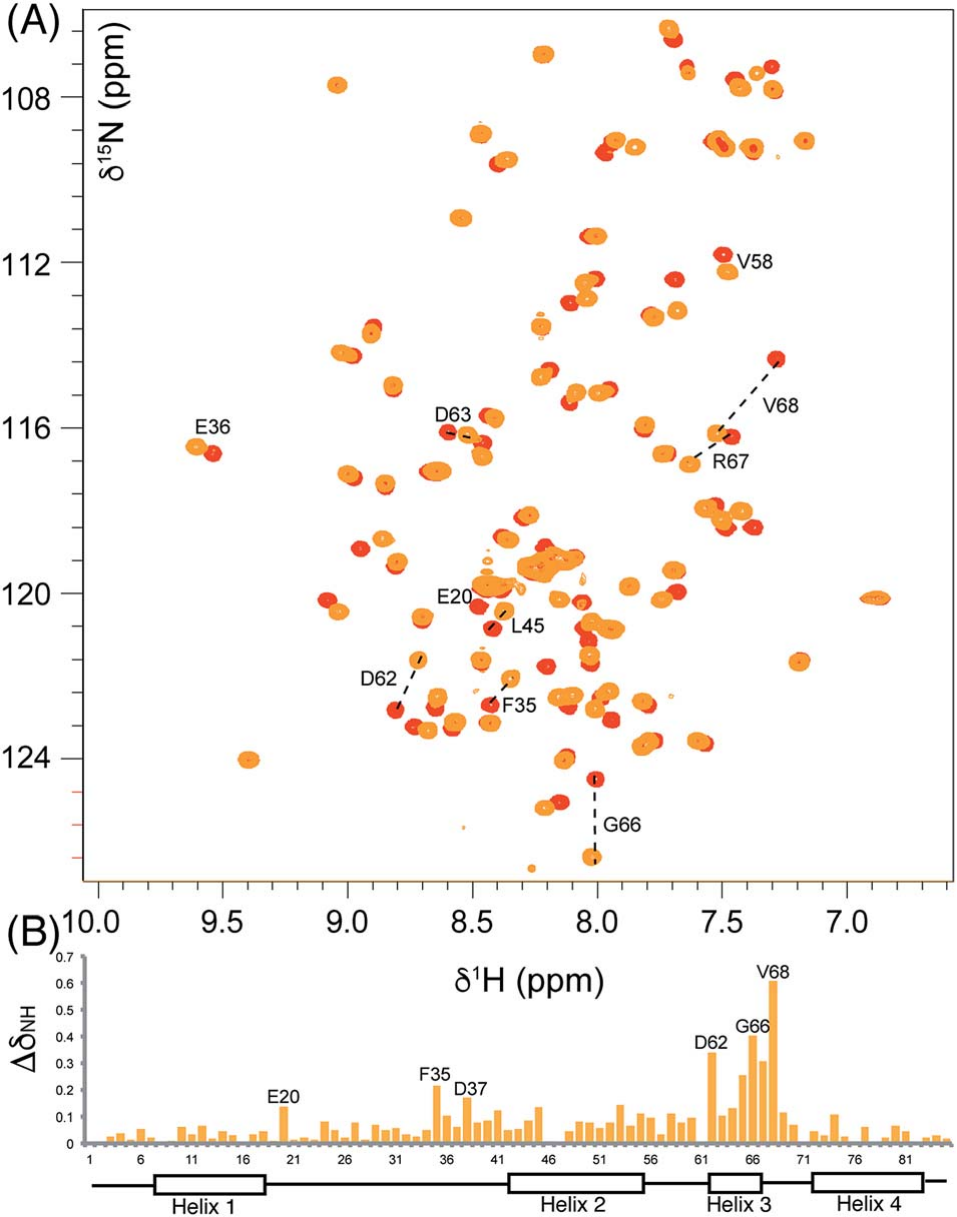
Comparison of the ^1^H–^15^N HSQC spectra of holo and aromatic act ACPs and chemical shift perturbations. (A) The overlaid ^1^H–^15^N HSQC of holo (red) and aromatic (orange) act ACPs. (B) Δ*δ*_NH_ between holo and aromatic act ACPs shown in column format where the height is proportional to the difference in ppm.

Initially, a comparison of ^1^H–^15^N HSQC spectra of holo and 5-phenyl-3-oxo-pentyl ACP **7** (Fig. 2A) revealed several significant chemical shift perturbations (CSPs) involving helix two and helix three (Fig. 2B) as observed previously with buried non-polar and polar linear chains.^11^ This indicated that the aromatic moiety might be tightly associated with the ACP. A sample of uniformly ^13^C, ^15^N labelled 5-phenyl-3-oxo-pentyl ACP was prepared and the solution structure solved using triple resonance techniques combined with ^13^C- and ^15^N-edited NOESY spectra. The solution structure of **7** was calculated using a total of 2424 NOE restraints and 55 TALOS *φ*/*ψ* dihedral angle restraints (see Table S1† for structural quality and statistics).^16,17^ Two-dimensional ^13^C, ^15^N filtered NOESY spectra that distinguish NOEs arising from ^12^C and ^13^C bound protons were also employed to identify and confirm specific ligand–ligand and protein–ligand interactions (Fig. S1†). These experiments yielded 21 ligand–ligand interactions and 22 protein–ligand NOEs, which defined the fold of the 4′-PP derivative and interactions between the aromatic ring and buried hydrophobes with the ACP.

**Fig. 3:**
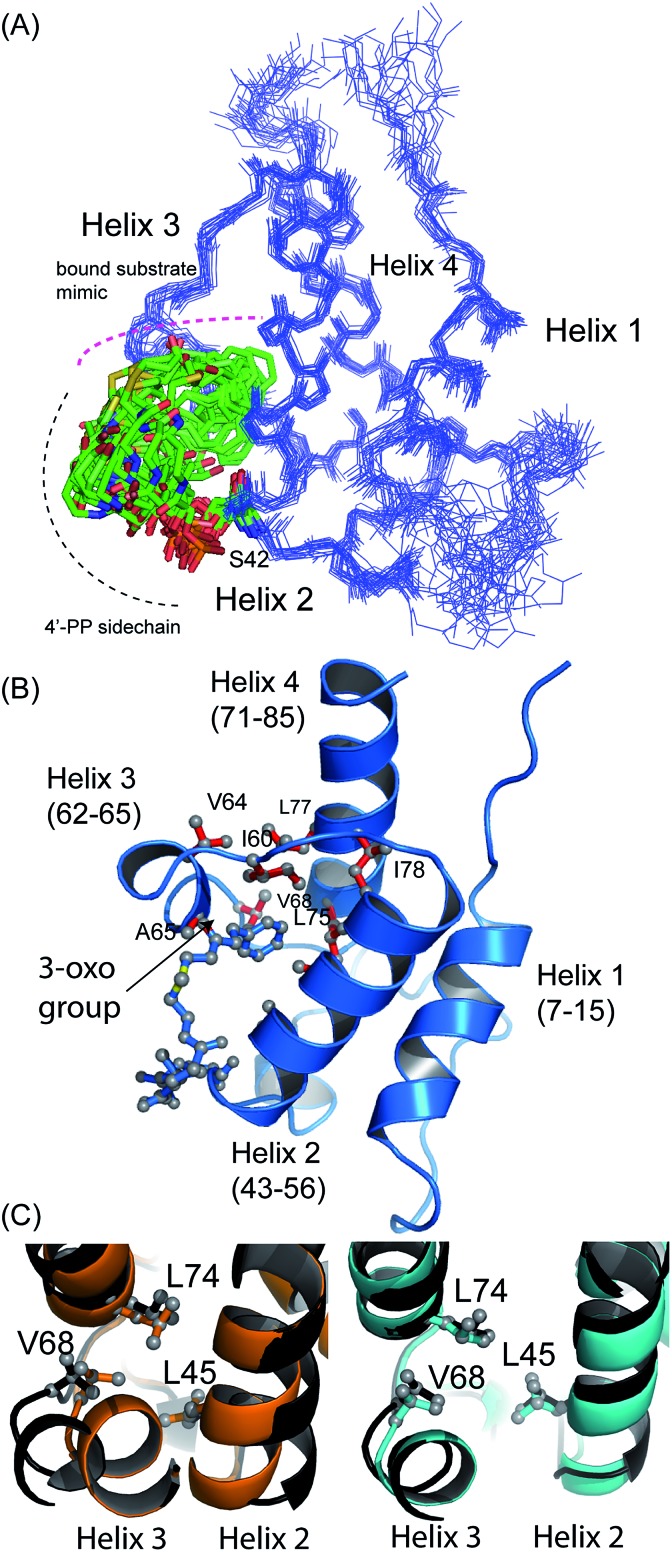
Structural ensembles and representative model for 5-phenyl-3-oxo-pentyl ACP **7**. (A) The ensemble of twenty structures rendered as a C_α_ trace. The 4′-PP derivative is represented in stick form and is buried in the core of the protein. (B) Ribbon image of the 5-phenyl-3-oxo-pentyl ACP **7** showing insertion of the phenyl moiety into the hydrophobic core of the protein. (C) Pairwise superimposed structures of holo (orange), octanoyl (cyan) and 5-phenyl-3-oxo-pentyl **7** (black) act ACPs. The residues, Leu45, Val68 and Leu74 are shown as sticks.

The structure of 5-phenyl-3-oxo-pentyl ACP **7** ¶
¶The ensembles of NMR structures and associated NMR chemical shifts have been deposited with the protein database and BioMagResBank: 5-phenyl-3-oxo-pentyl ACP (2MVV and rcsb104108), 3,7-dioxo-octyl ACP (2MVU and rcsb104107). revealed a characteristic four-helical bundle topology (Fig. 3A). The hydrophobic core is formed by Leu10 and Leu14 (helix 2), Leu45 and Leu52 (helix 2) and Leu74, Ile78 and Leu82 (helix 4) and Phe35, Ile38, Tyr40 and Ile60 on the inter-helical loops. Due to the relatively low number of protein to 4′-PP NOEs, the 4′-PP portion of the derivatised side-arm appears flexible in the final ensemble. Conversely, the phenyl ring is buried to the same degree in each of the calculated models (Fig. 3A) residing approximately parallel to the helical axis of helix three and equidistant to helix two and three across the ensemble. The angle with which the phenyl ring points into the cavity is however much less well defined and a distribution of conformers is observed. Although the phenyl ring is completely buried in the protein cavity, the remaining backbone of the 5-phenyl-3-oxo-pentyl derivative is largely unprotected and the 3-keto group is blocked at the entrance of the cavity and lies at the surface of the protein (Fig. 3B).

Acylation of act ACP with hydrophobic derivatives drives a conformational change of helix three^11^ that opens an internal cavity lined by residues Leu45, Met46, Thr48, Ala49, Ile60, Ala65, Val68 and Leu74. Likewise these residues form a cavity for docking the phenyl ring that also includes Val64, Leu77 and Ile78 due to the larger bulk of this aromatic group *versus* a linear hydrophobic chain. The methyl groups of Leu45, Val68 and Leu74 all show NOEs to the phenyl hydrogens and form the base of a cavity that binds the phenyl ring *via* numerous hydrophobic interactions. The side chains of Leu45 and Val68 adopt a single conformation in all twenty of the calculated models whereas a single conformation is observed in seventeen of the models for the side chain of Leu74.

Comparison with octanoyl ACP revealed that both octanoyl and 5-phenyl-3-oxo-pentyl ACP **7** are in the open conformations necessary for docking large hydrophobic side chains with similarities in amino acid side-chain conformations. The open cavity induced by the phenyl ring was, however, the largest we have observed to date at 222 Å^3^ compared to 188 Å^3^ in octanoyl act ACP. The backbone *φ* angle of the hinge residues Ile60 and Val68 provides an indirect readout of the conformational change induced in helix three and reflects the expansion of the cavity. The *φ* angle for Ile60 was measured at –94° ± 6, similar to those of hexanoyl and octanoyl act ACPs (–94° ± 2 and –94° ± 3, respectively)^11^ whereas the *φ* angle for Val68 was shifted to –108° ± 8 compared to –82 ± 2° in butyryl ACP, –86 ± 2° in hexanoyl ACP and –92 ± 4° in octanoyl ACP. The change in this latter value in particular further opens the internal cavity and the extra displacement around the C-terminal of helix three allows act ACP to open and bind the bulkier phenyl ring.

### Synthesis of 6-(3-hydroxyphenyl)-3-oxo-hexyl (phenolic) act ACP 16 and 3,7-dioxo-octyl ACP **20**

Having demonstrated the internal cavity of act ACP is able to expand and absorb a single phenyl group, we next determined whether incorporation of a greater degree of polarity would be accommodated in the same way or drive an alternative binding mode. To achieve this the phenolic CoASH derivative **15** was synthesised to explore whether the introduction of a single hydroxyl group on the aromatic ring would reduce or enhance binding to the ACP. Secondly an extended linear chain in the form of a 3,7-dioxo-octyl group was conjugated to CoASH to give **19**. This extended our previous studies to longer intermediates and tested whether increased chain length enhanced ACP binding, potentially through the selective binding of the hydrophobic face of the polyketide.

For the phenolic ACP **16**, protection of commercially available 3-bromophenol as silyl ether **8** followed by a Sonogashira cross-coupling with 3-butyn-1-ol gave alcohol **9** in 76% yield over the two steps (Scheme 2A). Reduction of the alkyne to **10** followed by Swern oxidation gave aldehyde **11** which on treatment with vinylmagnesium bromide gave allylic alcohol **12**. Acid hydrolysis of the TBS ether gave **13**, which was oxidised with MnO_2_ to give the desired enone **14** albeit in low yield. The reactivity of this enone towards Michael addition with thiols was first tested using propanethiol to avoid unnecessary consumption of expensive CoASH. This reaction proceeded cleanly within 1 h by NMR and could also be carried out in water without loss of reactivity or selectivity. These conditions were then used to tether the enone to CoASH to give **15** and then to the ACP to yield **16**.

For the synthesis of **20** (Scheme 2B), treatment of the δ-hexalactone with vinylmagnesium bromide gave alcohol **17**. Dess–Martin oxidation yielded enone **18** which subsequently reacted *via* a Michael addition with CoASH to yield **19** and then coupled to the ACP to give **20**.

**Fig. 4:**
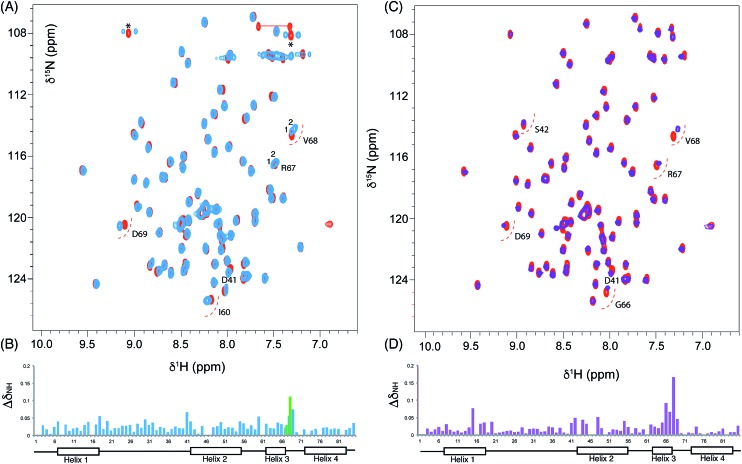
Chemical shift perturbations derived from 3,7-dioxo-octyl ACP **20** and 6-(3-hydroxyphenyl)-3-oxo-hexyl ACP **16**. (A) Comparison of the ^1^H–^15^N HSQC spectra of holo (red) and 3,7-dioxo-octyl ACP **20** (blue) and (B) associated CSPs. Blue bars represent the CSPs of the major form observed whilst a green bar is also shown when two different conformers were observable (*i.e.* for R67 and V68). (C) Comparison of the ^1^H–^15^N HSQC spectra of holo (red) and 6-(3-hydroxyphenyl)-3-oxo-hexyl holo ACP **16** (purple) collected at 600 MHz and (D) associated CSPs.

**Fig. 5:**
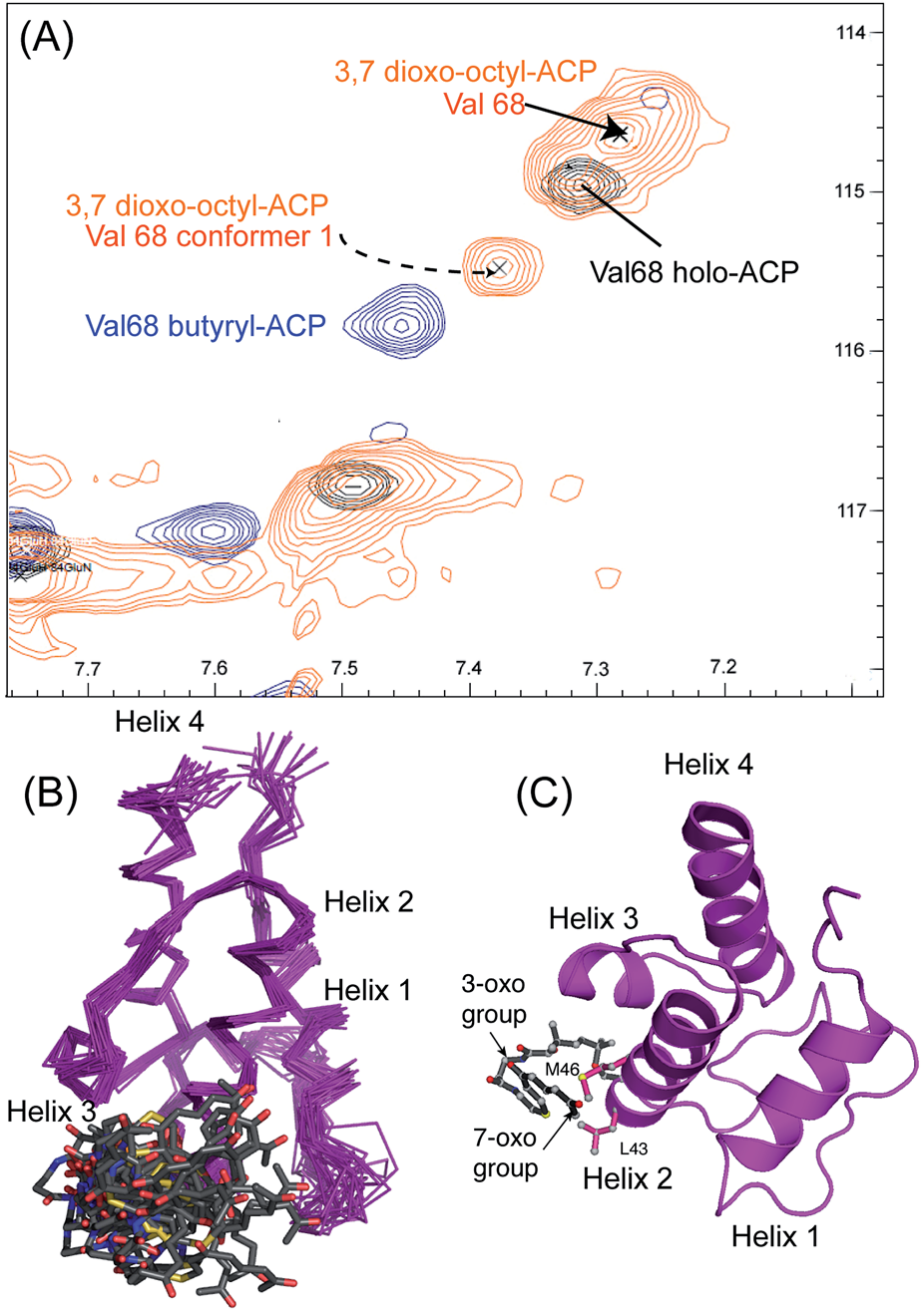
Conformational sampling induced in the 3,7-dioxo-octyl ACP structures. (A) Expansion of the ^1^H–^15^N HSQC spectra plotted at lower contour levels than Fig. 4 comparing 3,7-dioxo-octyl ACP (orange), butyryl ACP (blue) and holo ACP (black). A second conformer observed in 3,7-dioxo-octyl ACP indicates sampling of a partially opened structure approaching that of butyryl ACP. (B) Ensemble of twenty structures of 3,7-dioxo-octyl ACP. (C) A single conformer of 3,7-dioxo-octyl ACP showing how intra-molecular 4′-PP interactions fold the polar intermediate back along helix two.

We utilised ^1^H–^15^N HSQC spectra to probe the interaction of both polar analogues with act ACP (Fig. 4A and B). We observed CSPs relative to holo-ACP for both analogues although the magnitude of the changes was reduced compared to the CSPs observed with 5-phenyl-3-oxo-pentyl ACP. This indicated that that sequestration within the cavity of the ACP and concomitant conformational changes in the protein were reduced, suggestive of a transient, possibly surface interaction with the protein. In fact a second conformation was observed for 3,7-dioxo-octyl ACP **20** (Fig. 5A) with chemical shifts of the minor form suggestive of shallow binding similar to that observed in butyryl act ACP^11^. Based on these observations we calculated the three-dimensional structure of 3,7-dioxo-octyl ACP by solution state NMR (Fig. 5B, see ESI Table S2†). This structure corresponded to the major conformer (with the smallest CSPs) as the minor conformations were not in sufficient concentrations for NOEs to be observed. For the major conformer we observed numerous NOEs within the 4′-PP side-arm (methylenes H6′′ and H9′′, see ESI Fig. S2†) which were not observed in holo-ACP but were also detected when the ACP was previously derivatised with shorter non-polar side-chains.^11^ In addition 4′-PP – protein NOEs were observed from Leu 43 and Met 46. No NOEs could be observed to the 3,7-dioxo-octyl group. Nonetheless the NOEs that were observed were sufficient to define the topology of the 4′-PP sidearm which extends away from the ACP then folds back on itself to bring the thioether linkage close to helix two. This association is likely to be stronger in the two other sub-states that show greater protein CSPs and suggest the ACP is sensitised to the polar cargo and confers modest sequestration of the substrate, most likely by binding weakly along the charged groove between helices two and three (at the surface, rather than buried). Weak NOEs were observed previously from a short triketide analogue and emodic acid,^13^ a surrogate for a more highly elaborated actinorhodin scaffold, to residues in helix two in act ACP. In addition binding has been detected in act ACP bearing longer polyketide mimics utilising carbonyl replacement. These studies combined molecular modeling and CSPs observed in helix three to suggest that residency of the polyketide mimic on the ACP increased in line with the length and degree of cyclisation of the polyketide.^18^

The similarity of the observed CSPs between the phenolic ACP **16** and the 3,7-dioxo-octyl ACP **20** led us to conclude that a similar binding mode would be observed for **16** and therefore we did not calculate a three-dimensional structure. The weak interaction with the phenolic derivative is however in stark contrast to the burial of the phenyl ring indicating that the introduction of the single hydroxyl group is sufficient to drive an exclusion of the phenolic group from within the binding cavity to a weaker interaction at the surface of the ACP.

**Fig. 6:**
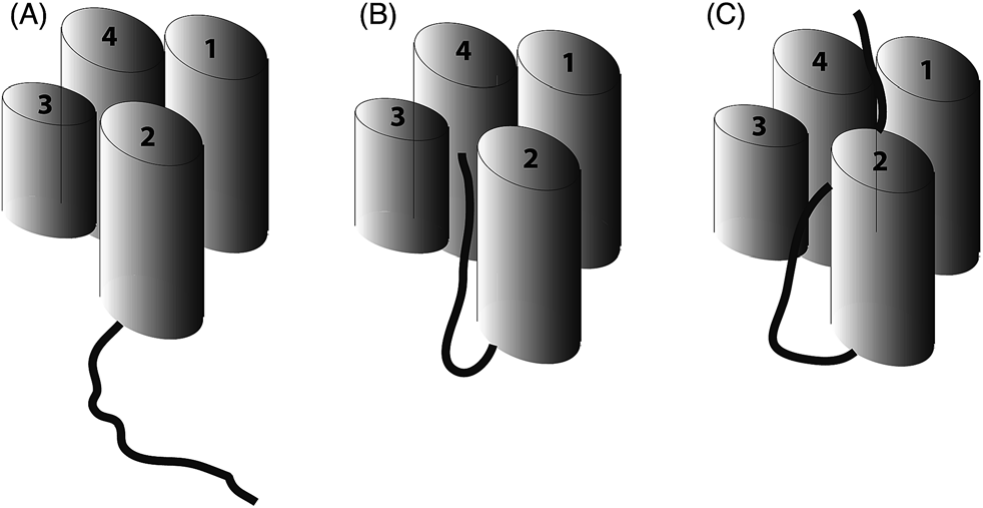
Schematic representation of ACP (four helices as cylinders) and attached intermediate (black). (A) A type I FAS ACP ACP that does not sequester a fatty acid chain (B) type II (and most likely type I) polyketide ACPs show partial sequestration of intermediate mimics and (C) full sequestration of saturated fatty acid chains by type II ACPs.

The work of our group and others^18,19^ now provides clear evidence that polyketide ACPs sequester their intermediates through a binding mode that is quite distinct from that employed in fatty acid synthesis (Fig. 6). Why is fatty acid sequestration important in fatty acid biosynthesis? The binding cavity employed by type II FAS ACPs offers some protection from thioester hydrolysis which has been shown to increase in rate as the fatty acid exceeds ∼14 carbons in length and outgrows the capacity of the ACP cavity although *in vitro* C_18_-ACP preparations of these ACPs are stable for many days.^20^ Burial of the fatty acid requires ‘chain-flipping’ of the fatty acid from the dynamic ACP to the more static fatty acid synthase enzymes (KS, KR, DH and ER) where neither association can be too stable as it would hinder the next flip.^21^ By comparison, a model for the initial steps of polyketide biosynthesis involves the stabilisation of the polyketide by the catalytic KS/CLF during chain elongation to a precisely controlled chain length.^22^ The active site is tube-like and located at the interface of the two domains.^23^ The elongated geometry of the site may assist in preventing unwanted spontaneous cyclisations and the amphipathic nature of this region may complement the nascent polyketide with suitable stabilising interactions, *e.g.* select ketide groups of the intermediate may be enolised to interact with two backbone carbonyls and a carboxylate side chain present in the amphipathic tunnel. In addition, a water molecule in the active tunnel may also play a role in stabilising the polyketide intermediate by donating a hydrogen bond to a ketone group. Once the mature polyketide has reached the correct length, a suitably strong interaction with the ACP might trigger dissociation with the ACP providing a suitably polar, but shallow cavity. The partial sequestration of the polyketide may be sufficient to act as a solvent shield during transit to the KR. Within the complex milieu of the bacterial cell, the KR may of course be associated with the KS/CLF as a non-covalent complex requiring minimal translocation.^24^ Our results taken together with related studies^18^ suggest that the ACP offers a degree of protection but in the absence of the next (*i.e.* KR) synthase component, the formation of SEK4b occurs, *via* an off-pathway C10–C15 cyclisation. In the normal pathway, the correctly C7–C12 cyclised product is then reduced to alcohol **2** by the KR and blockage of biosynthesis at this point leads to the formation of mutactin.^25^

## Conclusions

It appears therefore that a polyketide ACP protects a more polar intermediate through an association at the surface of the protein between the cleft formed by helix two and three rather than deep burial of the non-polar chains observed in fatty acid synthesis. The association is strong enough to induce multiple conformers in the ACP when the polyketide chain is of sufficient length and contains polar atoms. These polar intermediates do not however induce the large cavity opening we observed when act ACP was derivatised with non-cognate fatty acids and the role, if any, of this potential internal hydrophobic pocket remains elusive. The observed association of the 4′-PP chain with the ACP would still therefore require a degree of chain flipping involving dissociation from the ACP surface prior to insertion into the active site of the next synthase partner in the biosynthetic sequence. This may be more easily or rapidly achieved in the case of a surface associated polyketide intermediate *versus* a strongly bound fatty acid within a FAS ACP.
