# Supplementary material for: Recognition of extended linear and cyclised polyketide mimics by a type II acyl carrier protein
Source: Chem Sci. 2015 Dec 10;7(3):1779–85. doi: 10.1039/c5sc03864b (PMC5595124; doi:10.1039/c5sc03864b)
Supplement: Supplementary file 1 [file SC-007-C5SC03864B-s001.pdf]

Electronic Supplementary Material (ESI) for Chemical Science.
This journal is © The Royal Society of Chemistry 2015

Supporting Information

Recognition of extended linear and cyclised polyketide mimics by a Type II acyl carrier protein.

Xu Dong, Christopher D. Bailey, Christopher Williams, John Crosby, Thomas J. Simpson, Christine L. Willis* and Matthew P. Crump*

Table of Contents

Experimental

General experimental procedures and syntheses of compounds **4-19**

Figures

Figure S1 Strips extracted from a ^{13}C-edited NOESY-HSQC three-dimensional spectrum of 5-phenyl-3-oxo-pentyl ACP

Figure S2 F1f F2f NOEs of 5-phenyl-3-oxo-pentyl and 3,7-dioxo-octyl act ACPs

Table S1 Structural statistics and quality indicators for the 5-phenyl-3-oxo-pentyl ACP

Table S2 Structural statistics and quality indicators for 3,7-dioxo-octyl act ACP

Supporting Spectra

Mass spectra of CoASH derivatives and modified ACPs

NMR spectra of novel compounds

Experimental Section

General

Manipulations involving air-sensitive materials were carried out on a vacuum line under $N_{2\,(g)}$, employing standard Schlenk techniques. All glassware was flame-dried before use as standard. Dry solvents were purchased (*Aldrich*, *Fluka*) or obtained by passage through an *Anhydrous Engineering* drying column. Other reagents requiring purification where indicated were done so according to Purification of Laboratory Chemicals (D. D. Perrin & W.L.F Armarego, 3rd Edition, Butterworth Heinemann, 1988). Solvents for extraction and chromatography were technical grade.

Purchased chemicals (*Aldrich*, *Acros*, *Fluka*) were used as received (unless otherwise stated). Flash chromatography was conducted using *Fluorochem* silica gel 60 (0.040 - 0.063). Eluting solvent used as indicated in the text. TLC was conducted with 0.25 mm *Merck* silica gel 60 F254 on aluminium plates, using solvent systems indicated in the text, visualising at 254 nm and developed using standard $KMnO_4$ dip with gentle heating.

All IR spectra were obtained as thin film using *Perkin-Elmer Spectrum One* apparatus; peaks are reported in cm^{-1} with the following intensities: s (strong, 70 – 100 %), m (medium, 30 – 70 %), w (weak, 1 – 30 %). NMR spectra were obtained from *Jeol Eclipse (400 MHz)*, *Delta (270 MHz)*, *Lambda 300 (MHz)*, *Delta 400, Varian VNMRS 400* or *Varian 500* instruments. ^{1}H NMR chemical shifts δ (ppm) are reported relative to residual solvent. ^{13}C NMR shifts δ (ppm) are reported relative to deuterated solvent. Multiplicities are indicated as *s* (singlet), *d* (doublet), *t* (triplet), *q* (quartet), *quin* (quintet), *br* (broadened), *app.* (apparent) or *m* (multiplet, when multiplicity is complex). Coupling constants, *J*, are reported in Hz. MS (EI, CI, HRMS) were conducted by the University of Bristol Mass Spectrometry Service using *Fisons Autospec* instruments. HPLC was performed using a Gemini C18 reverse phase column (100x4.6mm, 5 micron, Phenomenex) using an ÄKTA Purifier (GE Healthcare) at a flow rate of 1ml/min.

For protein accurate mass measurement, samples were denatured prior to analysis by nano-ESI MS. Solutions were prepared using an existing protocol for simultaneous desalting and denaturation by interaction with C4 chromatographic resin[1]. Nano-ESI MS analyses were performed using a QSTAR XL (ABSciex) equipped with a NanoMate™ (Advion Biosciences) chip-based nano-ESI source.

5-Phenyl-3-hydroxy-pent-1-ene (4) 3-Phenylpropionaldehyde (0.67 ml) was cooled to 0 °C under nitrogen then vinyl magnesium bromide (1.6 M in THF, 3.13 ml) was added drop-wise. The mixture was stirred in an ice bath for ten minutes. Then, the reaction warmed to room temperature and stirred for two hours. The reaction was quenched by the addition of saturated ammonium chloride solution (10 ml) and extracted with ethyl acetate. The organic layer was combined, dried over magnesium sulfate the solvent was removed *in vacuo* to give a yellow oil. The crude extract was purified by silica chromatography (EtOAc: Petrol 1:3) giving the product **4** as an oil (0.46 g, 56 % yield). δ_H (270 MHz, $CDCl_3$) 1.75-1.90 (2H, m, 4-H_2) 2.71 (2H, t, *J* 7.3 Hz, 5-H_2); 4.12 (1H, q, *J* 6.5, 3-H) 5.14 (1H, dd, *J* 10.6, 1, 1-*H*H); 5.25 (1H, d, *J* 17.0, 1, 1-H*H*); 5.92 (1H, ddd, *J* 17.0, 10.6, 6.5 Hz, 2-H); 7.19-7.26 (5H, m, aromatic). Spectral data are in accord with the literature.[2, 3]

5-Phenyl-3-oxopent-1-ene (5). Alcohol **4** (0.23 g) was dissolved in in DCM (5 ml) under nitrogen at room temperature and Dess Martin periodinane (15 % w/w in DCM, 4.7 ml) was added. The solution slowly became cloudy as it was stirred at room temperature overnight. The solution was filtered through filter paper and the solvent removed *in vacuo*. The crude product was purified by silica chromatography (using ether: petrol) giving enone **5** as an oil (0.14 g, 61% yield). δ_H (270 MHz, $CDCl_3$) 2.80-3.0 (4H, m, 4-H_2 and 5-H_2), 5.82 (1H, dd, *J* 17.5, 10.2, 2-H), 6.21 (1H, dd, *J* 17.5, 1.5, 1-*H*H), 6.37 (1H, dd, *J* 17.5, 1.5, 1-H*H*) 7.19-7.26 (5H, m, aromatic); δ_C (100 MHz, $CDCl_3$) 29.9 (C-5), 41.3 (C-4), 126.2 (aromatic), 128.3 (aromatic), 128.4 (aromatic),

128.6 (aromatic), 136.6 (C-2), 140.8 (C-6), 199.6 (C-3). The spectroscopic data are in accord with the literature[4].

Preparation of 5-phenyl-3-oxo-pentyl Coenzyme A (6). An aqueous solution of Coenzyme A (CoASH) tri-lithium salt (100 mM, 50 μl) was mixed with a solution of enone **5** in acetone (100 mM, 75 μl) and vortexed for five minutes, followed by the addition of distilled water to make up the solution up to 1 ml. The solution was vortexed for a further five minutes and centrifuged for five minutes at 13000 rpm before purification by HPLC using conditions described in the general experimental details. This procedure was repeated until approximately 13.8 mg of the conjugate addition product **6** was obtained (60-70% yield). δ_H (600 MHz, D_2O) 0.81 (3H, s, CH_3), 0.94 (3H, s, CH_3), 2.45 (2H, t, *J* 6.5, 6''-H_2), 2.58 (2H, t, *J* 6.5, 9''-H_2), 2.67 (2H, t, *J* 6.5, SCH_2), 2.78 (2H, t, *J* 6.5, SCH_2CH_2), 2.84-2.85 (4H, m, CH_2CH_2Ph), 3.30 (2H, t, *J* 6.5, 8''-H_2) 3.46 (2H, t, *J* 6.5, 5''-H_2), 3.61 (1H, d, *J* 9.4, 1''-*H*H), 3.85 (1H, d, *J* 9.4, 1''-H*H*), 4.02 (1H, s, 3''-H), 4.26 (2H, m 5'-H_2), 4.61 (1H, m, 4'-H), 4.86-4.88 (2H, m, 2'-H, 3'-H), 6.20 (1H, d, *J* 5.5, 1'-H) 7.2-7.31 (5H, m, ArH), 8.41 (1H, s, 2-H), 8.67 (1H, s, 8-H); δ_C (150 MHz, D_2O) 20.9 (CH_3), 23.59 (CH_3), 27.5 (SCH_2), 31.9 (CH_2), 33.2 (C-9''), 38.1 (C-6''), 38.17 (C-5''), 41.27 (C-8''), 45.0 (CH_2), 46.2 (CH_2), 67.81 (C-5'), 74.7 (C-1''), 76.9 (C-3''), 77.1 (C-2', C-3'), 86.5 (C-4'), 90.20 (C-1'), 121.8 (C5), 128.9-131.3 (ArC), 145.2 (C-8), 147.40 (C-2), 147.8 (C-6), 151.4 (C-4), 176.7 (C-7''), 178.1 (C4''), 217.5 (CO). ESMS *m/z* observed, 928.10, calculated 927.75. ^{1}H-NMR assignments of the CoASH portion were checked for consistency with that of Pal and Bearne[5].

3-(*tert*-Butyldimethylsilyloxy)bromobenzene (8). Commercially available 3-bromophenol (3.46 g, 20 mmol, 1 eq.), imidazole (1.5 g, 22 mmol, 1.1 eq.) and DMAP (5 mg, cat.) were dissolved in dry DCM (80 ml) under N_2 and cooled to 0 °C. A solution of *tert*-butyldimethylchlorosilane (3.3 g, 22 mmol, 1.1 eq.) in dry DCM (20 ml) was added slowly. The reaction was warmed to RT and stirred for 2 h and progress followed by TLC. Water (100 ml) was added and the phases

separated. The organic phase was washed with sat NH_4Cl (100 ml), dried over magnesium sulfate and the solvent removed *in vacuo*. The crude product was purified by column chromatography (100% PE) to yield the title compound **8** as a colourless oil (5.12 g, 89%). R_f 0.45 (100% PE); δ_H (400 MHz, $CDCl_3$) 0.21 (6H, s, $SiMe_2$), 0.99 (9H, s, $SiC(CH_3)_3$), 6.77 (1H, m, ArCH), 7.02 (1H, m, ArCH), 7.09 (2H, m, ArCH). Spectroscopic data are in accord with that published in literature[6, 7].

4-(3-*tert*-Butyldimethylsilyloxyphenyl)but-3-yn-1-ol (9). Bromide **8** (1 g, 3.5 mmol, 1 eq.) and 3-butyn-1-ol (0.33 ml, 4.4 mmol, 1.25 eq.) were dissolved in Et_3N (10 ml) and cooled to 0 °C under nitrogen. $(PPh_3)_2PdCl_2$ (74 mg, 0.1 mmol, 3 mol%) and CuI (13.5 mg, 0.07 mmol, 2 mol%) were added and the solution stirred for 5 mins at 0 °C. After this time the reaction was heated 70 °C for 16 h. The reaction was cooled to RT, Et_2O (100 ml) was added and the organic phase washed with water (100 ml). The aqueous phase was further extracted with Et_2O (100 ml) and the combined organic extracts washed with brine (100 ml), dried over magnesium sulfate, filtered and the solvent removed *in vacuo* to yield a brown oil which was purified by column chromatography (1:1 PE:EtOAc) to yield alcohol **9** as an orange oil (0.833 g, 86%). R_f 0.5 (1:1 PE:EtOAc); δ_H (400 MHz, $CDCl_3$) 7.15 (1H, t, *J* 8.0, ArCH), 7.02 (1H, app. dt, *J* 8.0, 1, ArH), 6.90 (1H, m, ArH), 6.79 (1H, ddd, *J* 8.0, 2.5, 1, ArH), 3.82 (2H, app. q, *J* 6, 1-H_2), 2.70 (2H, t, *J* 6, 2-H_2), 1.82 (1H, t, *J* 6, OH), 0.99 (9H, s, tBu), 0.20 (6H, s, $SiMe_2$); δ_C (100 MHz, $CDCl_3$), 155.4 (C), 129.3 (CH), 124.9 (CH), 124.3 (CH), 123.2 (C), 120.3 (CH), 86.0 (C), 82.4 (C), 61.1 (CH_2), 25.6 (CH_3), 23.8 (CH_2), 18.2 (C), -4.4 (CH_3); IR (cm^{-1}), 3345 (br, w), 2955 (w), 2930 (w), 2859 (w), 1596 (m), 1574 (m), 1478 (m), 1291 (m), 1253 (m), 1192 (m), 876 (m), 828 (s), 779 (s); HRMS: *m/z* (CI), calculated for $C_{16}H_{25}O_2Si$ 277.1624 $[M+H]^+$, found 277.1618 $[M+H]^+$

4-(3-*tert*-Butyldimethylsilyloxyphenyl)butan-1-ol (10). Alcohol **9** (554 mg, 2 mmol, 1 eq.) and 10% Pd/C (55 mg, 10 wt%) were added to ethanol (20 ml) and stirred. Dissolved gases were removed by vacuum and H_2 introduced by balloon through a septum. The reaction was stirred

under a balloon pressure of H_2 for 2 h. Note: The reaction progression could not be followed by TLC as no R_f difference between SM and product so a small aliquot can be removed and analysed by ^{1}H NMR. On completion the flask was placed under vacuum and nitrogen introduced before being filtered carefully through Celite, ensuring that the Pd/C was not allowed to dry out. The filter cake was washed with CH_2Cl_2 and the filtrate concentrated *in vacuo* to yield alcohol **10** as a yellow oil (540 mg, 96%). δ_H (400 MHz, $CDCl_3$) 7.15-7.11 (1H, m, ArCH), 6.78 (1H, br. d, *J* 8, ArCH), 6.68-6.66 (2H, m, ArCH), 3.67 (2H, t, *J* 6.5, 1-H_2), 2.60 (2H, t, *J* 7.5, 4-H_2), 1.73-1.57 (4H, m, 2-H_2, 3-H_2), 1.23 (1H, br. s, OH), 0.99 (9H, s, tBu), 0.20 (6H, s, $SiMe_2$); δ_C (100 MHz, $CDCl_3$), 155.6 (C), 143.9 (C), 129.1 (CH), 121.4 (CH), 120.2 (CH), 117.4 (CH), 62.8 (CH_2), 35.5 (CH_2), 32.3 (CH_2), 27.4 (CH_2), 25.7 (CH_3), 18.2 (C), -4.4 (CH_3); IR (cm^{-1}), 3331 (br, w), 2930 (w), 2858 (w), 1603 (w), 1584 (w), 1484 (w), 1441 (w), 1272 (m), 1156 (m), 836 (m); HRMS: *m/z* (CI), calculated for $C_{16}H_{29}O_2Si$ 281.1937 $[M+H]^+$, found 281.1946 $[M+H]^+$

4-(3-*tert*-Butyldimethylsilyloxyphenyl)butanal (11). Alcohol **10** (534 mg, 1.9 mmol, 1 eq.) and DMSO (162 µL, 2.3 mmol, 1.2 eq.) were dissolved in dry CH_2Cl_2 (3 ml) and cooled to -78 °C under nitrogen. Oxalyl chloride (178 µL, 2.1 mmol, 1.1 eq.) was added slowly and stirred for 20 mins at -78 °C. Triethylamine (1.32 ml, 9.5 mmol, 5 eq.) was added and the reaction warmed to RT and stirred for 1 h and progress followed by TLC. On completion the reaction mixture was diluted with water (20 ml) and extracted with Et_2O (2 x 50 ml). The combined organic phases were washed with brine (20 ml), dried over magnesium sulfate, filtered and the solvent removed *in vacuo* to yield aldehyde **11** which was used without further purification (530 mg, quant). R_f 0.63 (2:1 PE:EtOAc); δ_H (400 MHz, $CDCl_3$) 9.77 (1H, t, *J* 1.5, 1-H), 7.15 (1H, app. t, *J* 8, ArCH), 6.77 (1H, br. d, *J* 8, ArCH), 6.70-6.66 (2H, m, ArCH), 2.61 (2H, t, *J* 7.5, 4-H_2), 2.45 (1H, dt, *J* 7.5, 1.5, 2-H_2), 1.95 (2H, app. quin, *J* 7.5 Hz, 3-H_2), 0.99 (9H, s, tBu), 0.20 (6H, s, $SiMe_2$); δ_C (100 MHz, $CDCl_3$), 202.3 (CHO), 155.7 (C), 142.7 (C), 129.3 (CH), 121.5 (CH), 120.2 (CH),

117.7 (CH), 43.1 (CH_2), 34.8 (CH_2), 25.7 (CH_3), 23.5 (CH_2), 18.2 (C), -4.4 (CH_3); IR (cm^{-1}), 2930 (w), 2859 (w), 1725 (w), 1602 (w), 1584 (w), 1484 (w), 1273 (m), 1253 (m), 1157 (m), 836 (s), 779 (s); HRMS: *m/z* (CI), calculated for $C_{16}H_{27}O_2Si$ 279.1780 $[M+H]^+$, found 279.1788 $[M+H]^+$

6-(3-*tert*-Butyldimethylsilyloxyphenyl)hex-1-en-3-ol (12). Aldehyde **11** (525 mg, 1.9 mmol, 1 eq.) in Et_2O (2.5 ml) was added slowly to stirring vinylmagnesium bromide solution (1M in THF, 2.1 ml, 2.1 mmol, 1.1 eq.) at -78 °C under nitrogen. On complete addition the reaction was warmed to RT and stirred for 1 h and progress followed by TLC. On completion the reaction mixture was quenched with sat. aq. NH_4Cl solution (20 ml) and water added to dissolve any precipitated salts. The aqueous solution was extracted with Et_2O (2 x 50 ml). The combined organics were washed brine (20 ml), dried over magnesium sulfate, filtered and the solvent removed *in vacuo* to yield allylic alcohol **12** as a colourless oil, which was used without further purification (540 mg, 94%)**.** R_f 0.5 (2:1 PE:EtOAc); δ_H (400 MHz, $CDCl_3$) 7.13 (1H, m, ArCH), 6.78 (1H, br. d, *J* 8, ArCH), 6.67-6.66 (2H, m, ArCH), 5.86 (1H, ddd, *J* 17, 10.5, 6.5, 2-H), 5.22 (1H, dt, *J* 17, 1.5, 1-H*H*), 5.11 (1H, dt, *J* 10.5, 1.5, 1-*H*H), 4.11 (1H, m, 3-H), 2.59 (2H, t, *J* 7.5, 6-H_2), 1.77-1.54 (4H, m, 4-H_2, 5-H_2), 1.46 (1H, br. d, *J* 4.5, OH), 0.99 (9H, s, tBu), 0.20 (6H, s, $SiMe_2$); δ_C (100 MHz, $CDCl_3$) 155.6 (C), 143.8 (C), 141.1 (CH), 129.1 (CH), 121.4 (CH), 120.2 (CH), 117.4 (CH), 114.7 (CH_2), 73.1 (CH), 36.5 (CH_2), 35.6 (CH_2), 27.0 (CH_2), 25.7 (CH_3), 18.2 (C), -4.4 (CH_3); IR (cm^{-1}), 3371 (br, w) 2930 (w), 2858 (w), 1603 (w), 1585 (w), 1485 (w), 1274 (w), 1157 (w), 840 (w); HRMS: *m/z* (ESI), calculated for $C_{18}H_{30}O_2NaSi$ 329.1907 $[M+Na]^+$, found 329.1917 $[M+Na]^+$

6-(3-Hydroxyphenyl)hex-1-en-3-ol (13) Allylic alcohol **12** (530 mg, 1.7 mmol, 1 eq.) was dissolved in EtOH (20 ml) and conc. aq. HCl (0.4 ml) added and the reaction stirred for 16 h at RT and progress followed by TLC. On completion the solvent was removed *in vacuo* and the

resultant residue purified by column chromatography (2:1 PE:EtOAc) to yield allylic alcohol **13** as a brown oil (246 mg, 75%). R_f 0.175 (2:1 PE:EtOAc); δ_H (400 MHz, $CDCl_3$) 7.17-7.13 (1H, m, ArCH), 6.76 (1H, br. d, *J* 8, ArCH), 6.67-6.65 (2H, m, ArCH), 5.87 (1H, ddd, *J* 17, 10.5, 6.5, 2-H), 5.23 (1H, dt, *J* 17, 1.5, 1-*H*H), 5.11 (1H, dt, *J* 10.5, 1.5, 1-H*H*), 4.91 (1H, br. s, ArOH), 4.13 (1H, m, 3-H), 2.60 (2H, t, *J* 7.5, 6-H_2), 1.80-1.50 (5H, m, 4-H_2, 5-H_2 & OH); δ_C (100 MHz, $CDCl_3$) 155.6 (C), 144.2 (C), 141.0 (CH), 129.5 (CH), 120.9 (CH), 115.3 (CH), 114.9 (CH_2), 112.7 (CH), 73.2 (CH), 36.4 (CH_2), 35.6 (CH_2), 26.9 (CH_2); IR (cm^{-1}), 3305 (br, w), 2939 (w), 1588 (m), 1456 (m), 1267 (m), 1155 (m), 908 (s), 730 (s); HRMS: *m/z* (ESI), calculated for $C_{12}H_{16}O_2Na$ 215.1042 $[M+Na]^+$, found 215.1051 $[M+Na]^+$

6-(3-Hydroxyphenyl)hex-1-en-3-one (14) Alcohol **13** (100 mg, 0.5 mmol, 1 eq.) was dissolved in THF (10 ml) and MnO_2 (1.8 g, 21 mmol, 40 eq.) added and the reaction stirred for 4 h at RT and progress followed by TLC. On completion the slurry was filtered through celite and the filter cake washed with DCM. The solvent was removed *in vacuo* yielding enone **14** as an orange oil (25 mg, 25%). R_f 0.325 (2:1 PE:EtOAc); δ_H (500 MHz, $CDCl_3$) 7.15 (1H, m, ArCH), 6.76 (1H, br. d, *J* 8, ArCH), 6.69-6.67 (2H, m, ArCH), 6.35 (1H, dd, *J* 17.5, 10.5, 2-H), 6.19 (1H, dd, *J* 17.5, 1, 1-*H*H), 5.82 (1H, dd, *J* 10.5, 1, 1-H*H*), 4.82 (1H, br. s, ArOH), 2.62 (2H, t, *J* 7.5, 4-H_2 or 6-H_2), 2.60 (2H, t, *J* 7.5 Hz, 4-H_2 or 6-H_2), 1.96 (2H, quin, *J* 7.5, 5-H_2); δ_C (125 MHz, $CDCl_3$), 200.7 (CO), 155.6 (CH), 143.5 (C), 136.5 (CH), 129.6 (CH), 128.1 (CH_2), 121.0 (CH_2), 115.4 (CH), 112.9 (C), 38.7 (CH_2), 34.9 (CH_2), 25.1 (CH_2); IR (cm^{-1}), 3357 (br, w), 2933 (w), 1667 (s), 1613 (s), 1598 (s), 1587 (s), 1455 (s), 1403 (m), 1273 (m), 1225 (m), 1155 (s), 968 (m), 781 (s), 695 (s); HRMS: *m/z* (CI), calculated for $C_{12}H_{15}O_2$ 191.1072 $[M+H]^+$, found 191.1076 $[M+H]^+$

6-(3-Hydroxyphenyl)hexyl-3-one CoASH (15) CoASH (20 μL, 100 mM) was mixed with enone **14** in acetone (20 μL, 100 mM) and 2 μL of potassium carbonate solution in H_2O (1 M).

The solution was initially cloudy and then vortexed for 5 minutes until clear to yield **15**, checked by ESMS mass (theoretical mass): 958.24 Da (957.78 Da) and was used directly in the coupling to ^{13}C, ^{15}N labelled-ACP.

7-Hydroxyoct-1-en-3-one (17) Dry THF (20 ml) was cooled to -78 ºC, followed by the addition of δ-hexalactone (0.62 ml, 5.5 mmol) under nitrogen. Magnesium vinyl bromide (3.2 ml, 1.6 M in THF) was then added drop-wise and reaction stirred at -78 ºC for 2 h under nitrogen. The reaction was washed with saturated NH_4Cl aqueous solution (10 ml) and extracted with EtOAc (3 x 20 ml) and the combined organic phase dried over magnesium sulfate and concentrated *in vacuo*. The crude product was purified through a silica column (EtOAc:petrol) to yield 7-hydroxy-oct-en-3-one **17** as a colourless oil (0.55 g, 3.9 mmol, 71%). δ_H (300 MHz, $CDCl_3$) 1.24 (3H, d, *J* 6.2, 8-H_3), 1.56-1.62 (2H, m, 5-H_2), 1.80-1.86 (2H, m, 6-H_2), 2.59 (2H, t, *J* 6.8, 4-H_2), 3.68 (1H, m, 7-H), 5.80 (1H, dd, *J* 10.2, 1.7, 1-*H*H), 6.19 (1H, dd, *J* 17.0, 1.7, Hz, 1-H*H*), 6.32 (1H, dd, *J* 17.0, 10.2, 2-H); δ_C (75 MHz, $CDCl_3$) 19.7 (C-5), 23.1 (C-8), 38.2 (C-6), 39.1 (C-4), 67.0 (C-7), 127.9 (C-1), 136.1 (C-2), 171.8 (C-3). Spectroscopic data are in accord with the literature.[8]

Oct-1-ene-3,7-dione (18). Dess Martin periodinane (0.58 g) was dissolved in CH_2Cl_2 (20 ml) under nitrogen, and this suspension was added drop-wise to a solution of alcohol **17** (0.14 g, 1mmol) in CH_2Cl_2 (20 ml) and stirred at room temperature for two hours. A cloudy solution formed during the addition. The reaction filtered through a silica column (EtOAc:petrol) and the solvent removed *in vacuo* to yield enone **18** as a colourless oil (0.11 g, 0.79 mmol, 79%). δ_H (300 MHz, $CDCl_3$) 1.94 (3H, s, 8-H_3), 1.75 (2H, m, 5-H_2), 2.47 (2H, t, *J* 7.2, 6-H_2), 2.60 (2H, t, *J* 7.2, 4-H_2), 5.75 (1H, dd, *J* 10.2, 1.5, 1-*H*H), 6.11-6.17 (1H, dd *J* 17.6, 1.5, 1-H*H*), 6.28 (1H, dd *J* 17.6 10.2, 2-H); δ_C (75 MHz, $CDCl_3$) 18.9 (C-5), 22.2 (C-8), 39.3 (C-4), 43.1 (C-6), 128.5 (C-1), 137.8 (C-2), 172.5 (C-3), 208.3 (C-7). Spectroscopic data are in accord with the literature.[9]

Synthesis and purification of 3,7-dioxo-octyl CoASH (19) An aqueous solution of CoASH

(100 mM, 50 μl) was added to enone **18** in acetone (100 mM, 65 μl) and mixed gently at room temperature for 2 hours, centrifuged and purified by HPLC using the conditions described in the general experimental details. ESMS mass (theoretical mass): 908.1 Da (907.71 Da) for **19**. δ_H (600 MHz, D_2O) 0.81 (3H, s, CH_3), 0.93 (3H, s, CH_3), 1.74 (2H, pent, *J* 7.3, $CH_2CH_2CH_2$), 2.16 (3H, s, $COCH_3$), 2.46 (2H, t, *J* 6.5, 6''-H_2), 2.52-2.56 (4H, m, H-4/H-6, $CH_2CH_2CH_2$), 2.64 (2H, t, *J* 6.5, 9''-H_2), 2.73 (2H, t, J 6.5, CH_2S), 2.81 (2H, t, *J* 6.5, $COCH_2$), 3.34 (2H, t, *J* 6.6, 8''-H_2), 3.47 (2H, t, *J* 6.5, 5''-H_2), 3.60 (1H, d, *J* 10.4, 1''-*H*H) 3.86 (1H, d, *J* 10.4, 1''-H*H*), 4.02 (1H, s, 3'-H), 4.26 (2H, m, 5'-H_2), 4.62 (1H, m, 4'-H), 4.86-4.88 (2H, m, 2'-H/3'-H), 6.20 (1H, d, *J* 5.5, 1'-H), 8.44 (1H, s, 2-H), 8.67 (1H, s, 8-H); δc (150 MHz, D_2O) 19.0 (CH_2), 20.9 (CH_3), 23.3 (CH_3), 27.6 (*S*-CH_2), 30.1 (CH_3), 33.20 (C9''), 38.2 (C5''), 38.0 (C6''), 41.3 (C8''), 44.6/44.8 (C4/C6), 44.8 (C2), 67.8 (C-5'), 74.6 (C-1''), 76.8 (C-2'/C-3' overlapped), 77.1 (C-3''), 86.3 (C-4'), 90.4 (C-1'), 121.22 (C-5), 145.2 (C-8), 147.1 (C-6), 147.4 (C-2), 151.40 (C-4), 176.7 (C-7''), 178.1 C-4'', 217.9 (CO), 221.0 (CO). ^{1}H-NMR assignments of the CoASH portion were checked for consistency with that of Pal and Bearne[5].

Preparation act ACP derivatives 7, 16 and 20 for NMR. 100mM CoASH derivative solution (H_2O, 2 μL) was added to a solution of unlabelled, ^{15}N or ^{13}C/^{15}N ACP solution (1.0 mM, H_2O, 10 μL) with 40 μM ACPS solution (5 μL in assay buffer) in a 1.5 mL Eppendorf tube. The protein solution was made up to 100 μL with 50 mM Tris, 10 mM $MgCl_2$ (pH 8.8) and incubated at 37 ºC for 24 hours. The progress of the assay was checked by mass spectrometry for 7 and 20 using unlabelled ACP (**7** [observed mass 9600 Da, expected 9600 Da], **20** [observed mass 9579.0, expected mass 9581.0 Da]. Preparation of ^{13}C, ^{15}N labelled ACP derivatives were subsequently prepared (^{13}C, ^{15}N labelled 5-phenyl-3-oxo-pentyl ACP **7** (expected mass for 100% ^{13}C/^{15}N incorporation 10101 Da, observed mass 10082 (~ 97% incorporation)) and ^{13}C, ^{15}N labelled 3,7-dioxooctyl ACP **20** (expected mass for 100 % ^{13}C/^{15}N incorporation 10081 Da, observed mass 10063 Da). ^{13}C, ^{15}N labelled **16** [observed mass 10118 Da, expected 10129.6 for 100% ^{13}C, ^{15}N

labelling]. Twenty reactions were performed simultaneously to obtain sufficient amounts of labelled ACP. After the reaction went to > 95% completion by mass spectrometry, the derivatised ACP was concentrated and buffer exchanged 5 times into 20 mM potassium phosphate (pH 5.5) using Centricon ultrafiltration unit (4 ml, Millipore, MWCO 3000 Da). The final samples (500 μL) were 1.7 mM, pH 5.4. NaN_3 (0.1 mM) in 92% H_2O/ 8% D_2O respectively.

Biological NMR data collection for acylated act ACP. All protein NMR experiments for act ACP were acquired at 25 °C on a Varian *INOVA* 600 MHz spectrometer and a cryoprobe equipped Varian VNMRS 600 MHz spectrometer. HNCACB, CBCACONH, CCONH, HCCONH, HNHA, HCCH-TOCSY, HNCO experiments were used to assign the backbone and side chain atoms. ^{15}N- edited and ^{13}C-edited NOESY datasets were acquired with 100 ms mixing times to generate distance restraints for structure calculation. For determining the interactions between isotopically labelled protein and unlabelled 4′-PP cofactor and derivatives, F_2f (i.e. ^{13}C signals removed in F_2) and F_1fF_2f-filtered TOCSY and NOESYs (150 ms mixing time) were acquired to detect the chemical shifts of the ligands and the protein-ligand contacts[10].

All the NMR data were processed using NMRPipe[11] spectral processing and analysis system, and the assignment and NOE data collection were analyzed using CCPN Analysis Version 2.1.3[12, 13]. Chemical shift differences were calculated using the following function[14]: $\Delta\delta_{avg}=[0.5\times(\Delta\delta H^2+0.2\times\Delta\delta N^2)]^{0.5}$, where ΔδH and ΔδN are the values of chemical shift changes in H and N dimensions, respectively.

The NOE peak list and assignments of protein atoms were exported using CCPNMR Format Converter[15] in NmrView format, which is accepted by the structure calculation programme. The φ and ψ dihedral angle restraints were analyzed using Torsion Angle Likelihood Obtained from

Shift and sequence similarity (TALOS). The chemical shift input for TALOS was generated using CCPNMR Format Converter[15]. Initially, classification of predictions was automatically determined by TALOS, and the borderline predictions were then manually assessed. Violations flagged by the structure calculation programme were either relaxed or removed from the TALOS restraints.

All structure calculations were performed using Ambiguous Restraints for Iterative Assignment (ARIA) version 1.2 or 2.2 coupled to CNS version 1.2. Topology and parameter files for the modified Ser42 were generated using the Dundee PRODRG server and added to the existing CNS files. The number of dynamics steps was increased over default values to 20000 and 16000 for the first and second cooling stages, respectively[16]. Initially, the chemical shift table, TALOS restraints and NOE peak lists were used as input data for structure calculation. After the first run, the problematic NOE restraints were flagged and checked manually in the spectra. Usually, the errors arose from noise, unaliased peaks and assignments falling out of the defined tolerances. The refined input was then used in the next run of structure calculations. This cycle was repeated until low energy and non-violated structures were obtained. In the last iteration of the calculation 100 structures were generated, and the 20 lowest energy structures were further refined in explicit water using the RECOORD protocol[17]. RMSD values were calculated using Molmol, and the structure qualities were assessed using the iCing web-server (https://nmr.cmbi.ru.nl/icing/iCing.html#file), which provided the assessments of the structure by WHAT IF and PROCHECK. Three-dimensional structures were analyzed and drawn using Pymol[18] Version 0.99 and 1.3. Internal hydrophobic cavities were predicted using the CastP web server[19]

Figure S1. **Strips extracted from a ^{13}C-edited NOESY-HSQC three-dimensional spectrum of 5-phenyl-3-oxo-pentyl ACP**. Representative strips from the ^{13}C methyl shifts for the groups of A, Ala49 Hβ*, B, Ala65 Hβ* and C, Val68 Hγ*. The protein-ligand NOEs are labeled with their assignments and chemical shifts of the corresponding ligand atoms. The numbering of atoms in the aromatic moiety of the 4'-PP derivative is shown below.

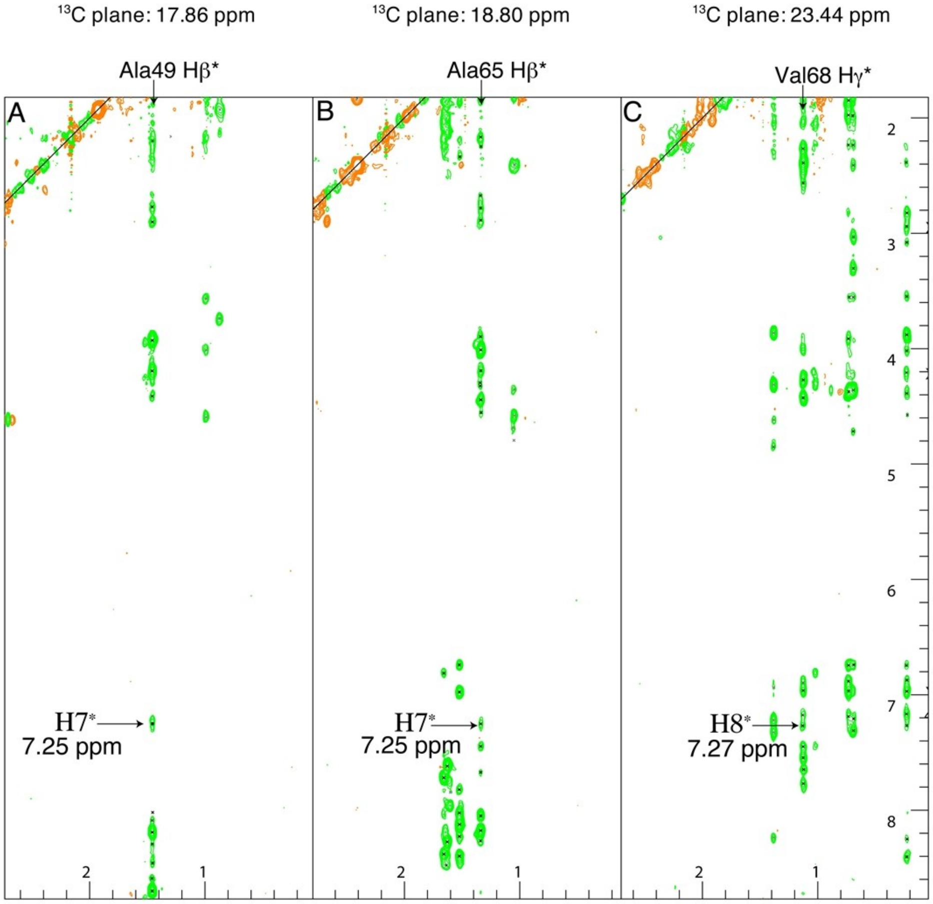

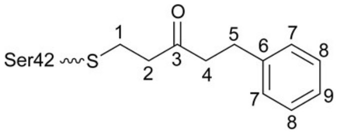

Figure S2. F1f F2f NOEs of a) 5-phenyl-3-oxo-pentyl and b) 3,7-dioxo-octyl act ACPs. In a) NOEs from 4’-PP methyl group protons (H30*/31*) to the atoms partway along the 4’-PP chain (shown with arrows), indicative that the 4'-PP side chain is bent back on itself and the aromatic group may be protected by the protein. Long-range NOEs are also observed in the F1fF2f spectrum of b) with the addition of weak NOEs from H30*/31* protons to H6” and H9”.

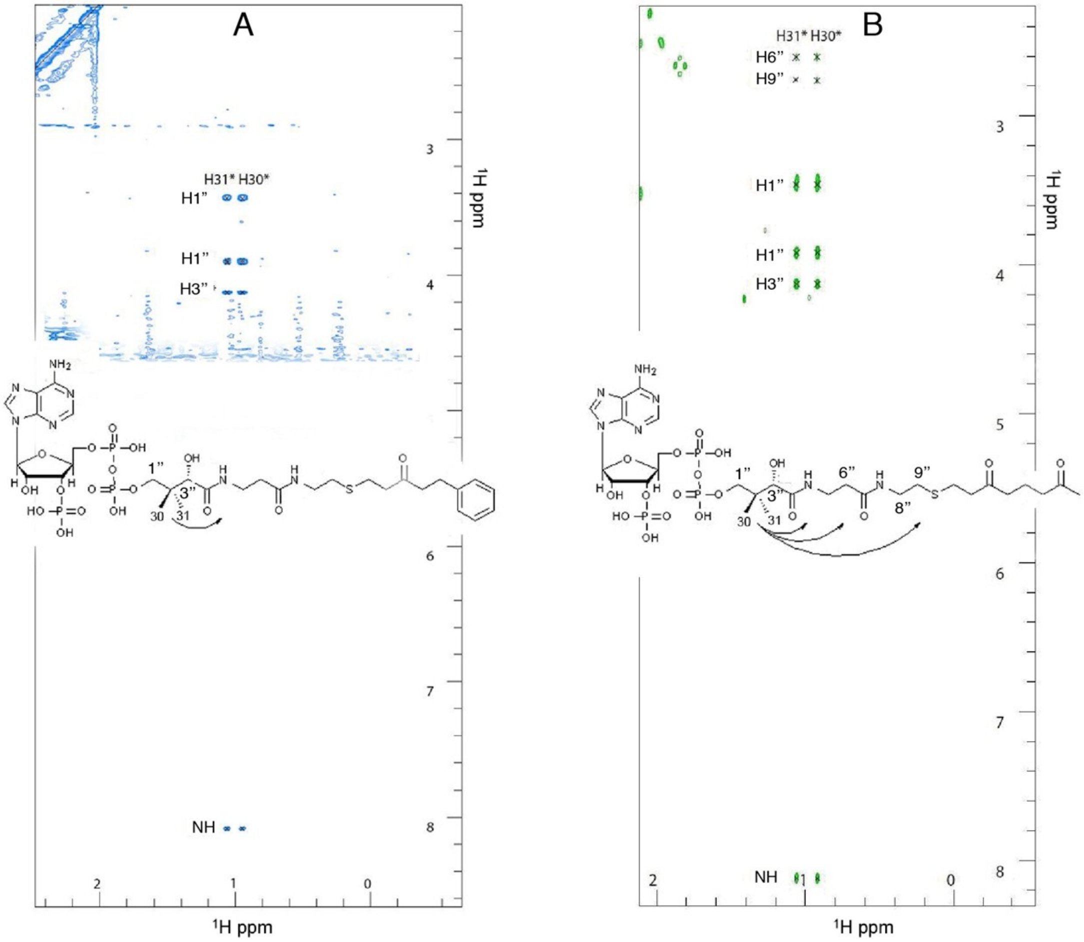

Table S1 Structural statistics and quality indicators for the 5-phenyl-3-oxo-pentyl ACP

**Number of restraints**	
total	2424 (29 per residue)
unambiguous	1987
ambiguous	437
intra residue	1010
sequential	540
medium range	344
long range	571
**Violation per structure**	
NOE > 0.5 Å	0
NOE > 0.3 Å	0
NOE > 0.1 Å	4
TALOS φ/ψ	0
**RMSD (Å)**	
well-ordered residues	0.46±0.06
all residues	0.95±0.15
**Ramachandran plot**	
most favoured	86.7%
additionally allowed	11.9%
generously allowed	1.1%
disallowed	0.3%
**Z-scores**	
2nd generation packing quality	-0.965
Ramachandran plot appearance	-2.393
$\chi 1/\chi 2$ rotamer normality	-1.009
Backbone conformation	-0.06

**Table S2** Structural statistics and quality indicators for 3,7-dioxo-octyl act ACP	
**Number of restraints**	
total	2308
unambiguous	1548
ambiguous	760
intra residue	883
sequential	515
medium range	334
long range	576
**Violation per structure**	
NOE > 0.5 Å	0
NOE > 0.3 Å	0
NOE > 0.1 Å	7
TALOS φ/ψ	0
**RMSD (Å)**	
well-ordered residues	0.49±0.06
all residues	1.00±0.12
**Ramachandran plot**	
most favoured	86.5%
additionally allowed	12.5%
generously allowed	0.7%
disallowed	0.3%
**Z-scores**	
2nd generation packing quality	-1.035
Ramachandran plot appearance	-2.449
χ1/χ2 rotamer normality	-1.366
Backbone conformation	0.189

Mass Spectra of 5-phenyl-3-oxo-pentyl CoASH **6** and 3,7-dioxo-octyl CoASH **20**.

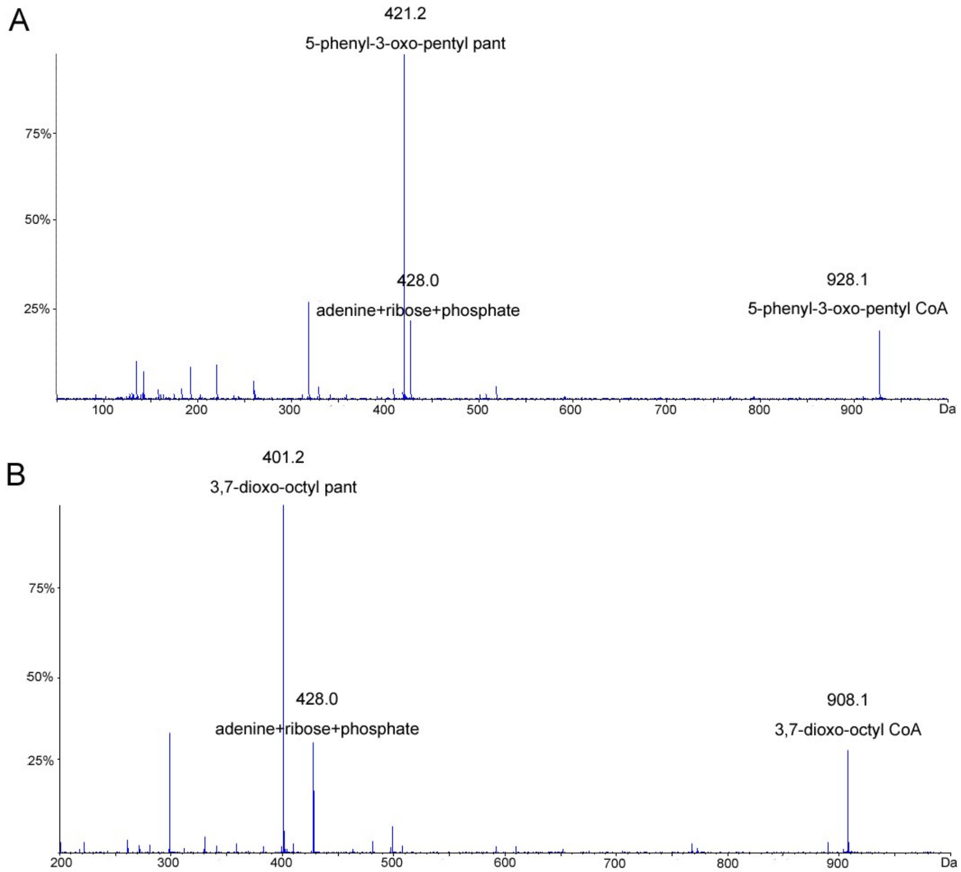

ESMS of 6-(3-hydroxyphenyl)hexyl CoASH **15.**

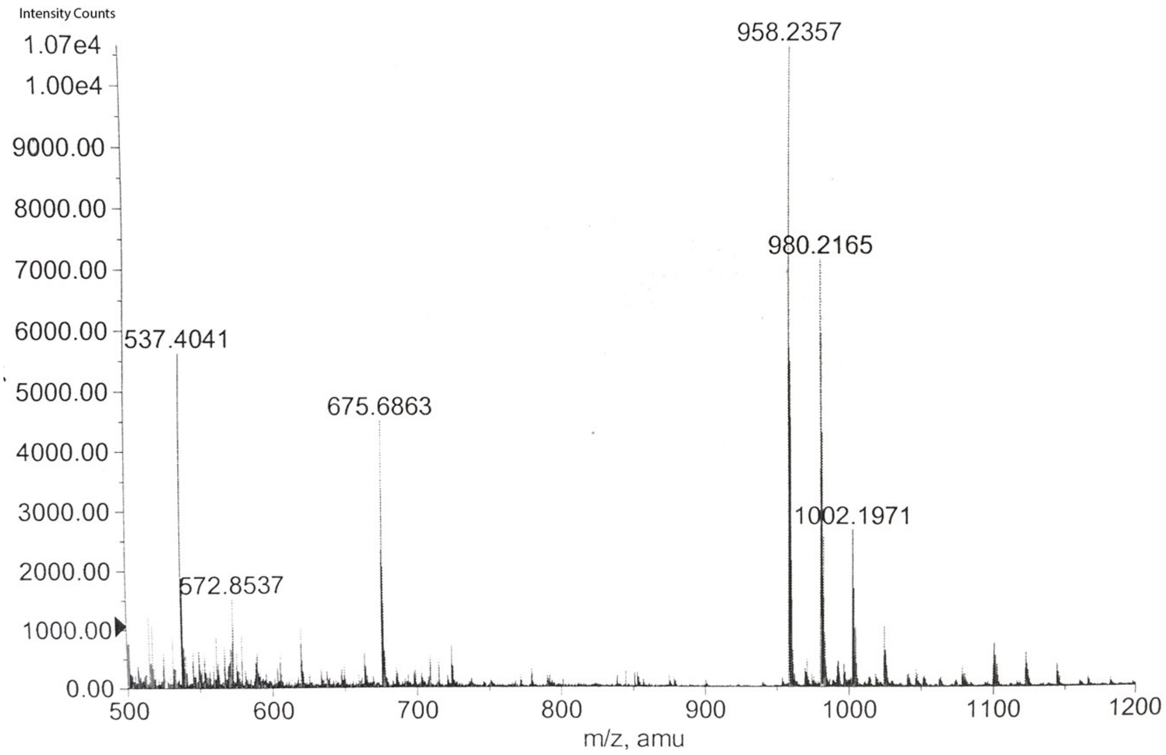

ESMS of 3,7-dioxo-octyl ACP.

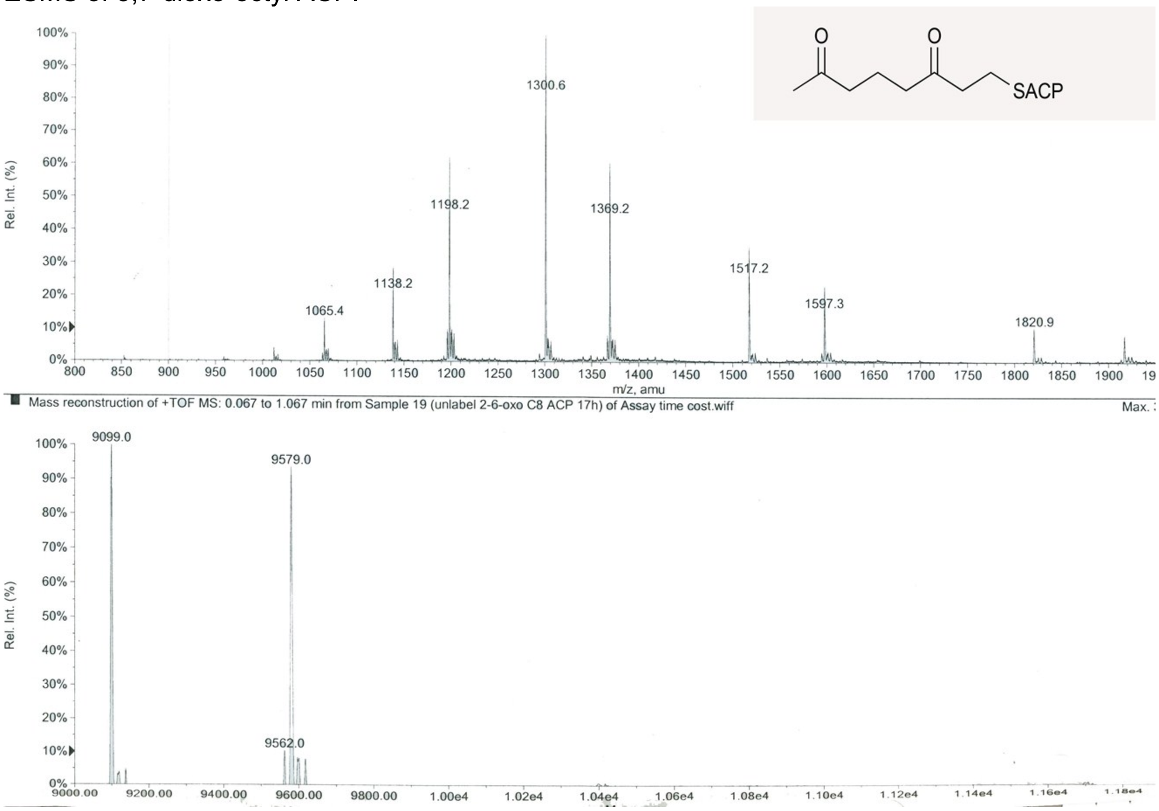

Time course showing production of 5-phenyl-3-oxo-pentyl ACP with time points taken at A) 6 hours, B) 23 hours and C) 31 hours

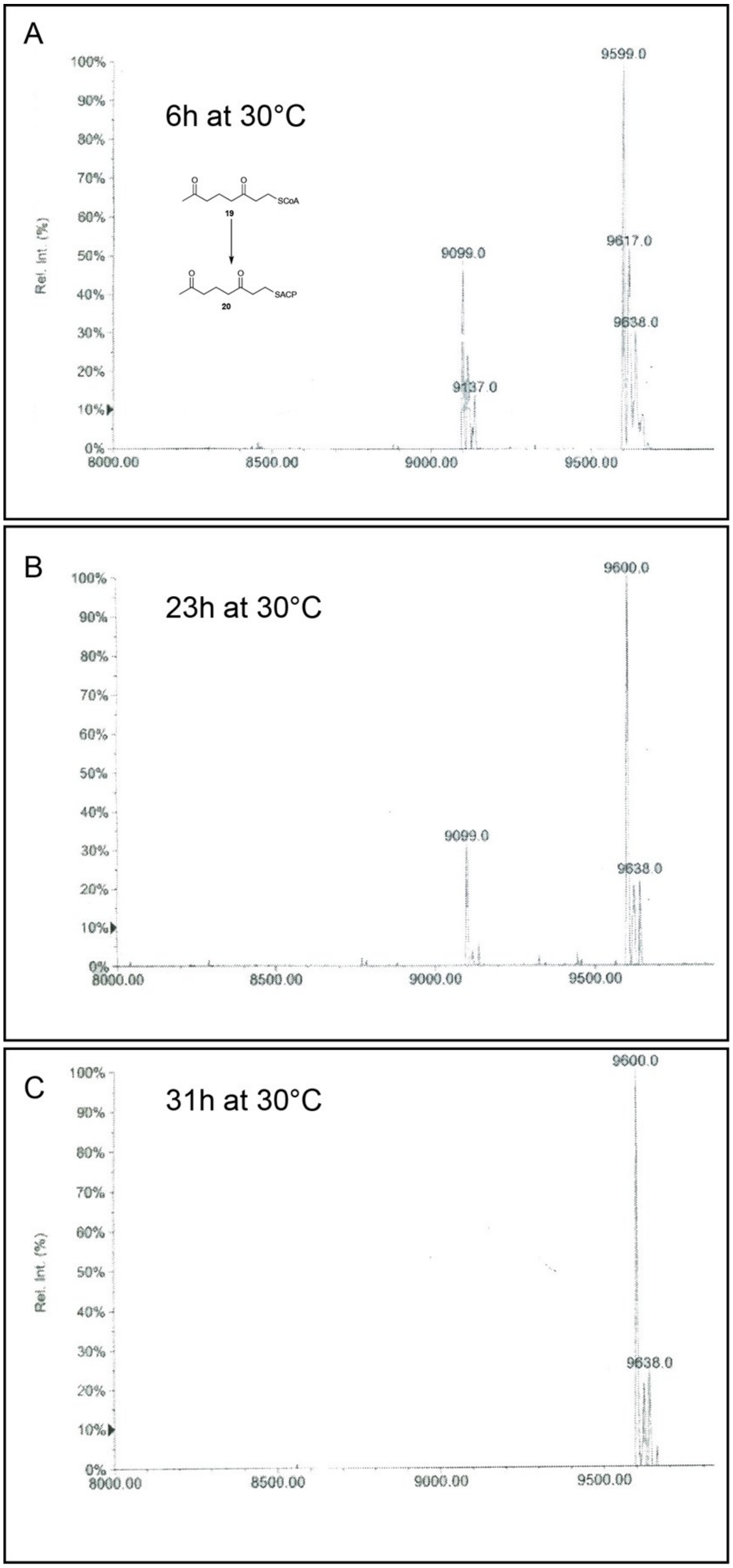

ESMS of (A) ^{13}C, ^{15}N labelled 5-phenyl-3-oxo-pentyl ACP **7** (expected mass for 100% 13C/15N incorporation 10101 Da) and (B) ^{13}C, ^{15}N labelled 3,7-dioxooctyl ACP **20** (expected mass for 100 % ^{13}C/^{15}N incorporation 10081 Da). The observed masses are fractionally lower indicating ~ 97% isotopic incorporation.

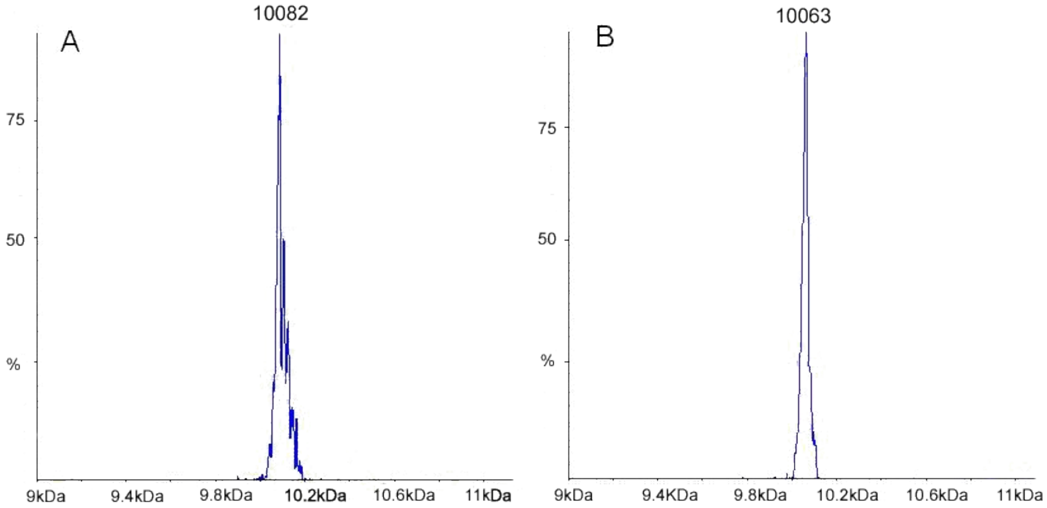

NMR Spectra of Novel Compounds.

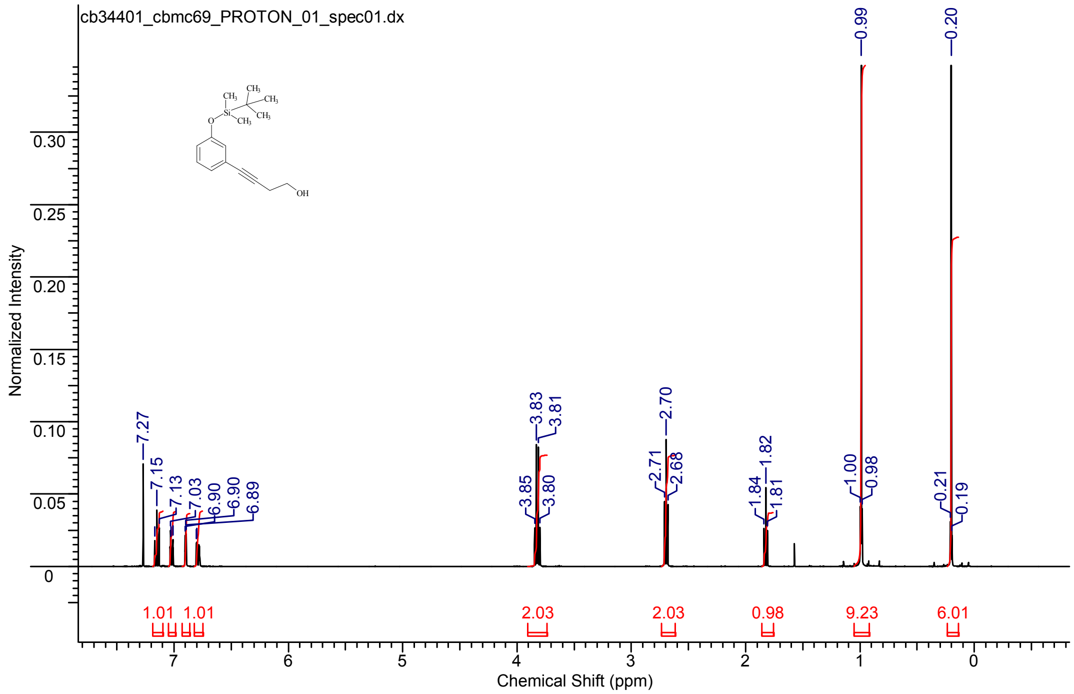

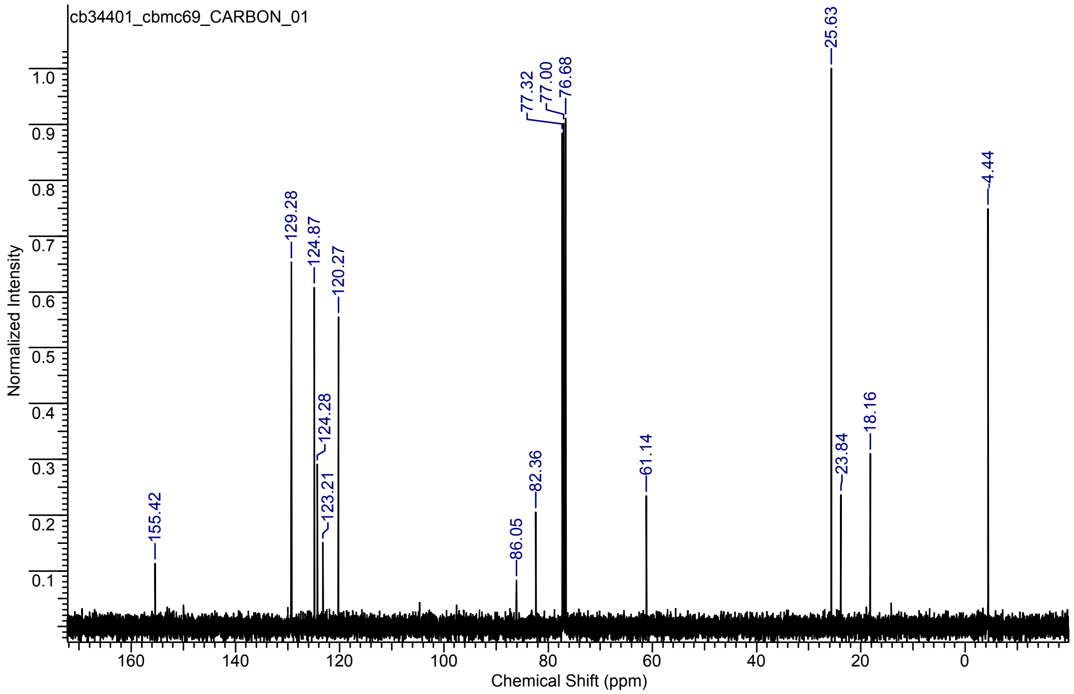

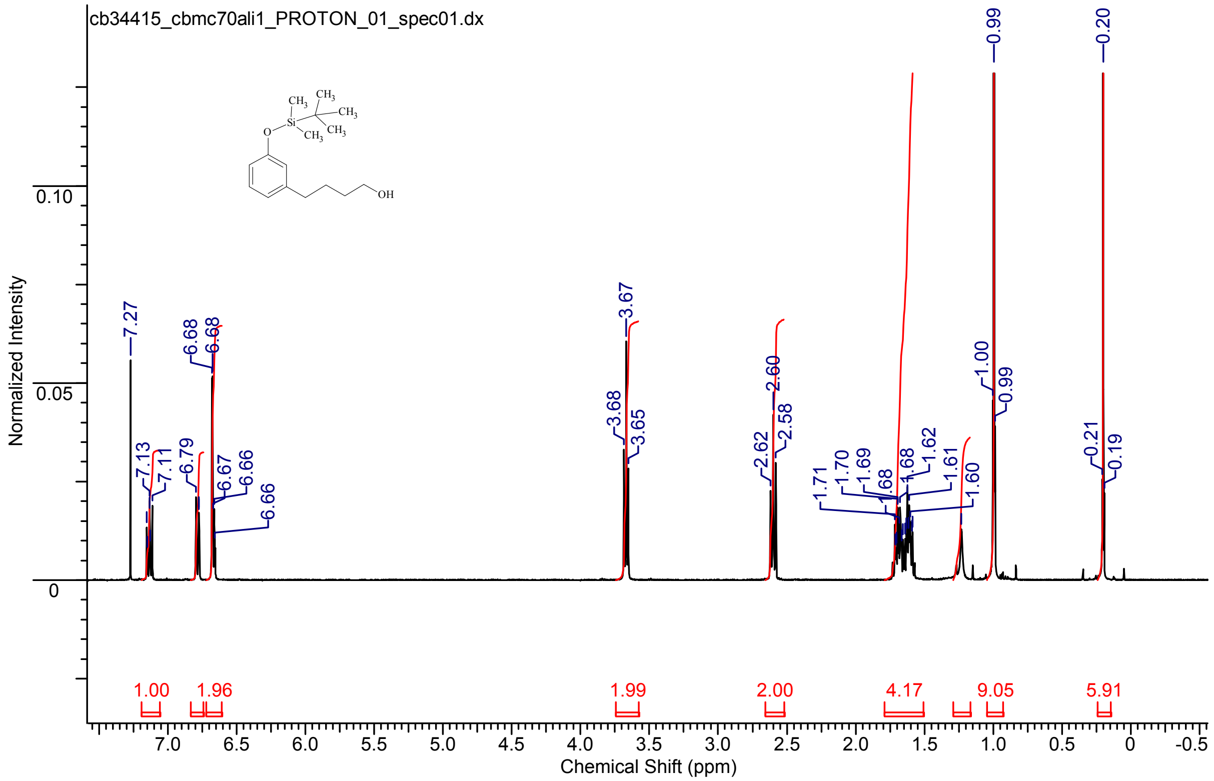
cb34415_cbmc70ali1_PROTON_01_spec01.dx
Normalized Intensity
Chemical Shift (ppm)
7.27
7.13
7.11
6.79
6.68
6.68
6.67
6.66
6.66
3.68
3.67
3.65
2.62
2.60
2.58
1.71
1.70
1.69
1.68
1.68
1.62
1.61
1.60
1.00
0.99
0.99
0.21
0.20
0.19
1.00
1.96
1.99
2.00
4.17
9.05
5.91
OH
O
Si
CH3
CH3
CH3
CH3
CH3

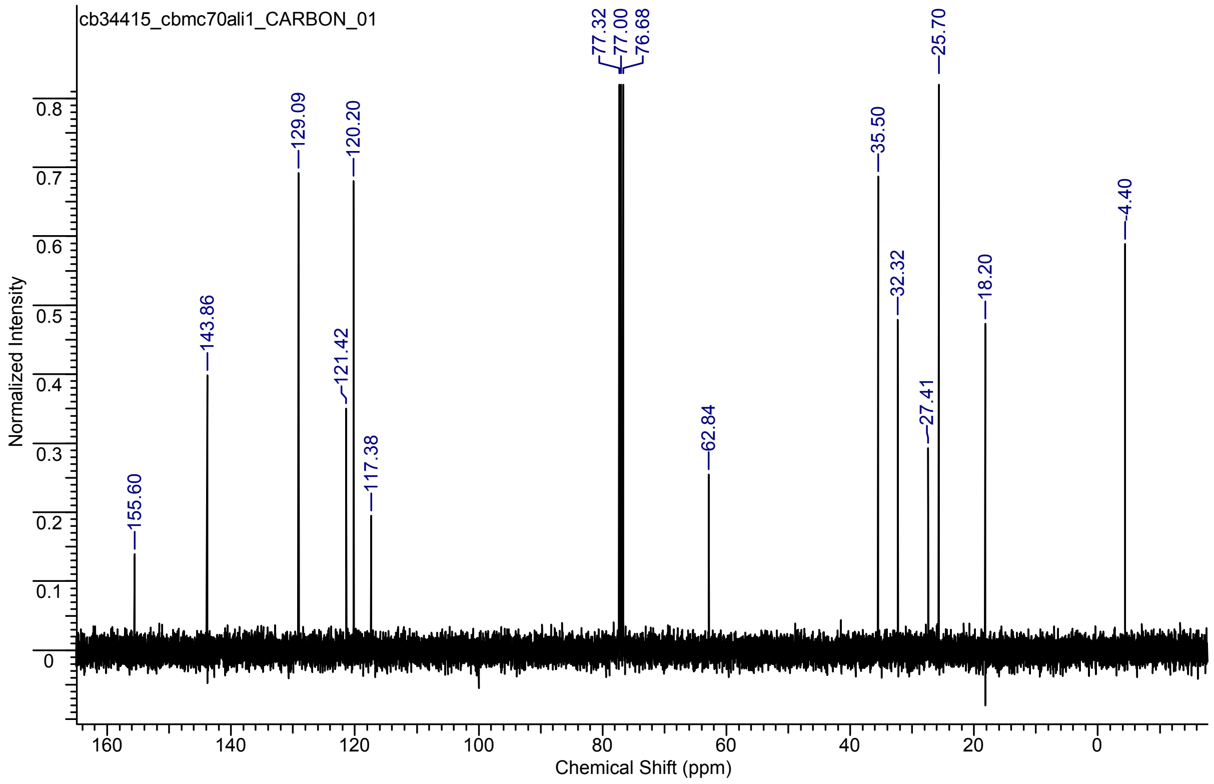
cb34415_cbmc70ali1_CARBON_01
Normalized Intensity
Chemical Shift (ppm)
155.60
143.86
129.09
121.42
120.20
117.38
77.32
77.00
76.68
62.84
35.50
32.32
27.41
25.70
18.20
-4.40

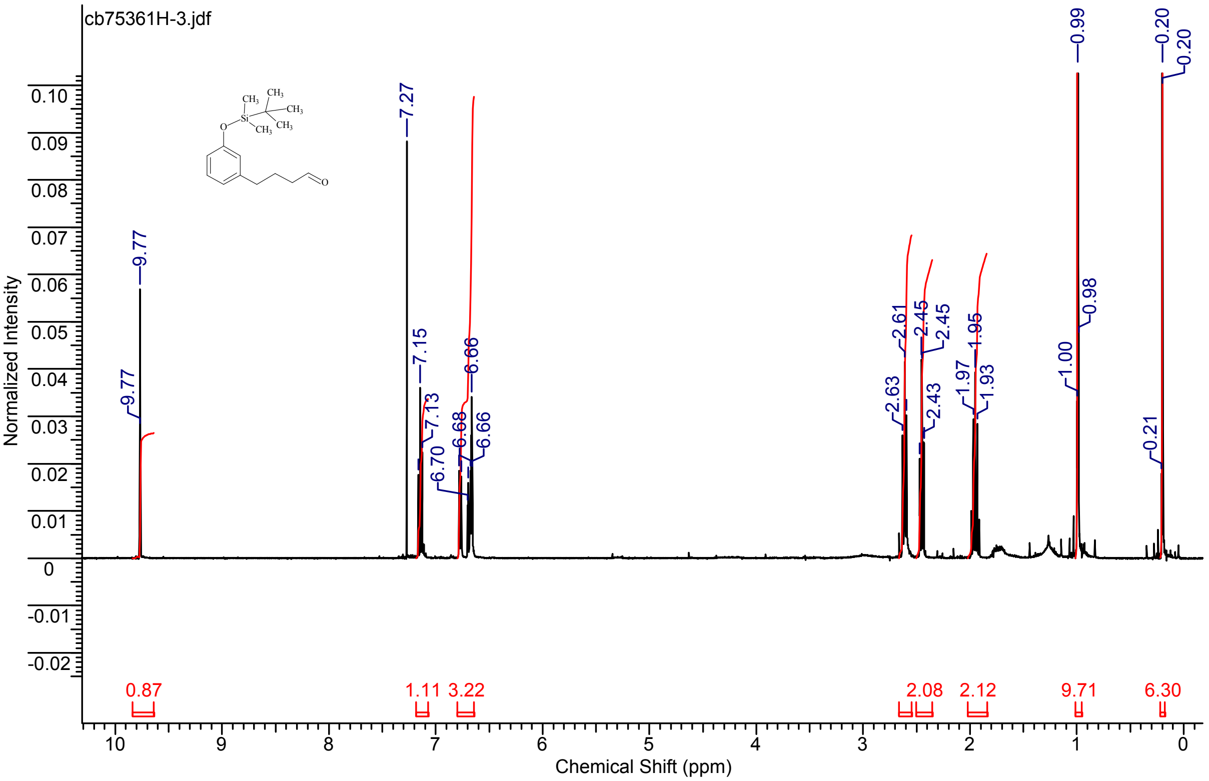
cb75361H-3.jdf
Normalized Intensity
Chemical Shift (ppm)
9.77
9.77
7.27
7.15
7.13
6.70
6.68
6.66
6.66
2.63
2.61
2.45
2.45
2.43
1.97
1.95
1.93
1.00
0.99
0.98
0.21
0.20
0.20
0.87
1.11
3.22
2.08
2.12
9.71
6.30

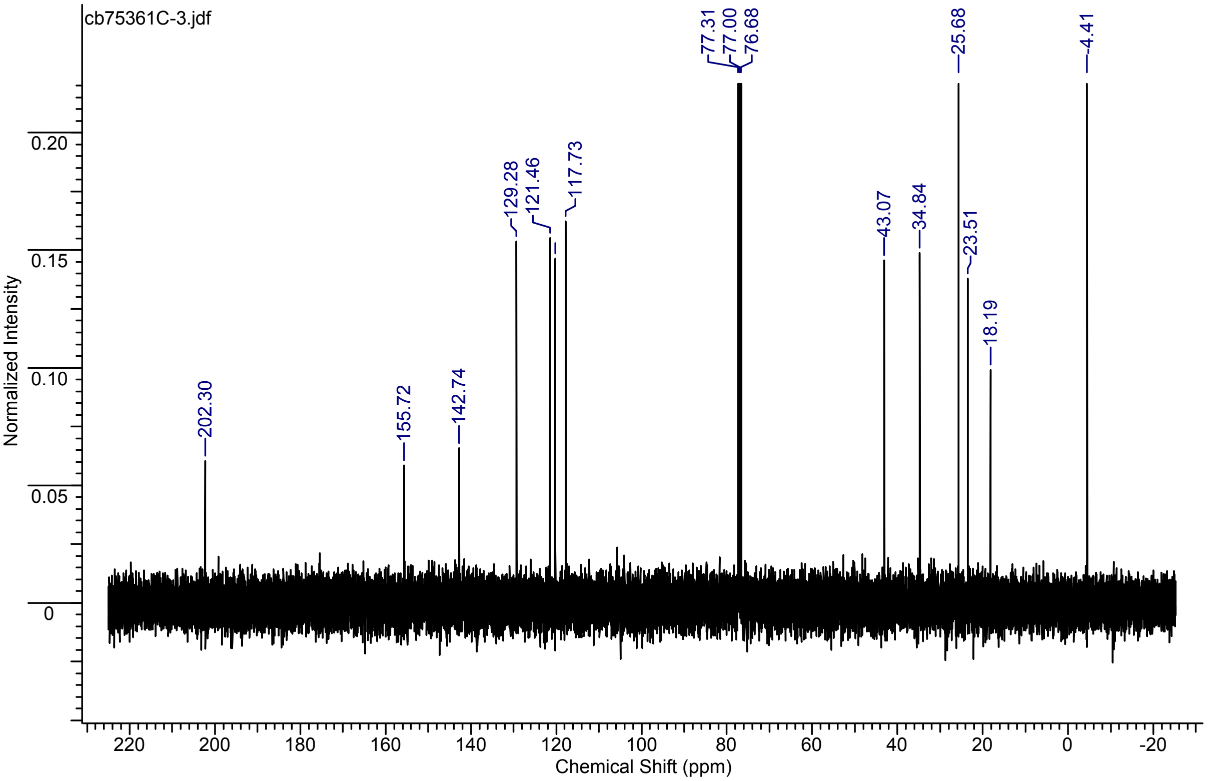
cb75361C-3.jdf
Normalized Intensity
Chemical Shift (ppm)
202.30
155.72
142.74
129.28
121.46
117.73
77.31
77.00
76.68
43.07
34.84
25.68
23.51
18.19
-4.41

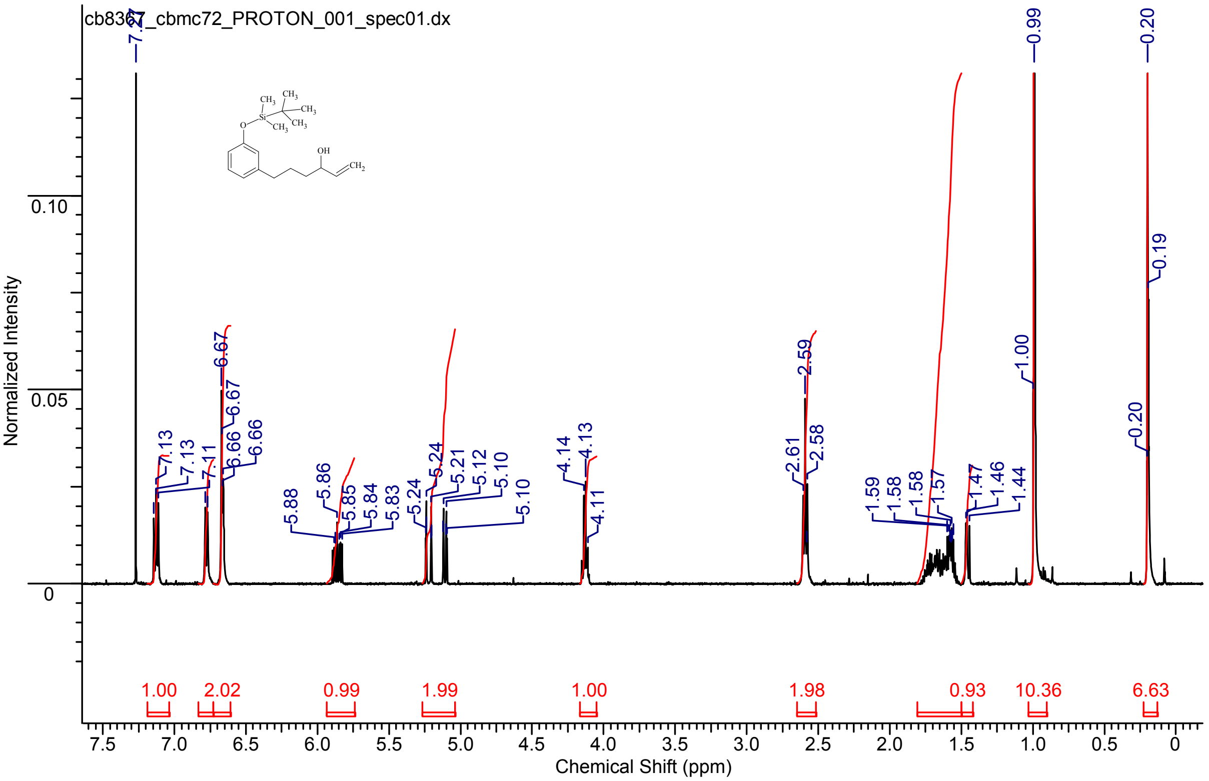
cb8367_cbmc72_PROTON_001_spec01.dx
Normalized Intensity
Chemical Shift (ppm)
1.00
2.02
0.99
1.99
1.00
1.98
0.93
10.36
6.63
OH

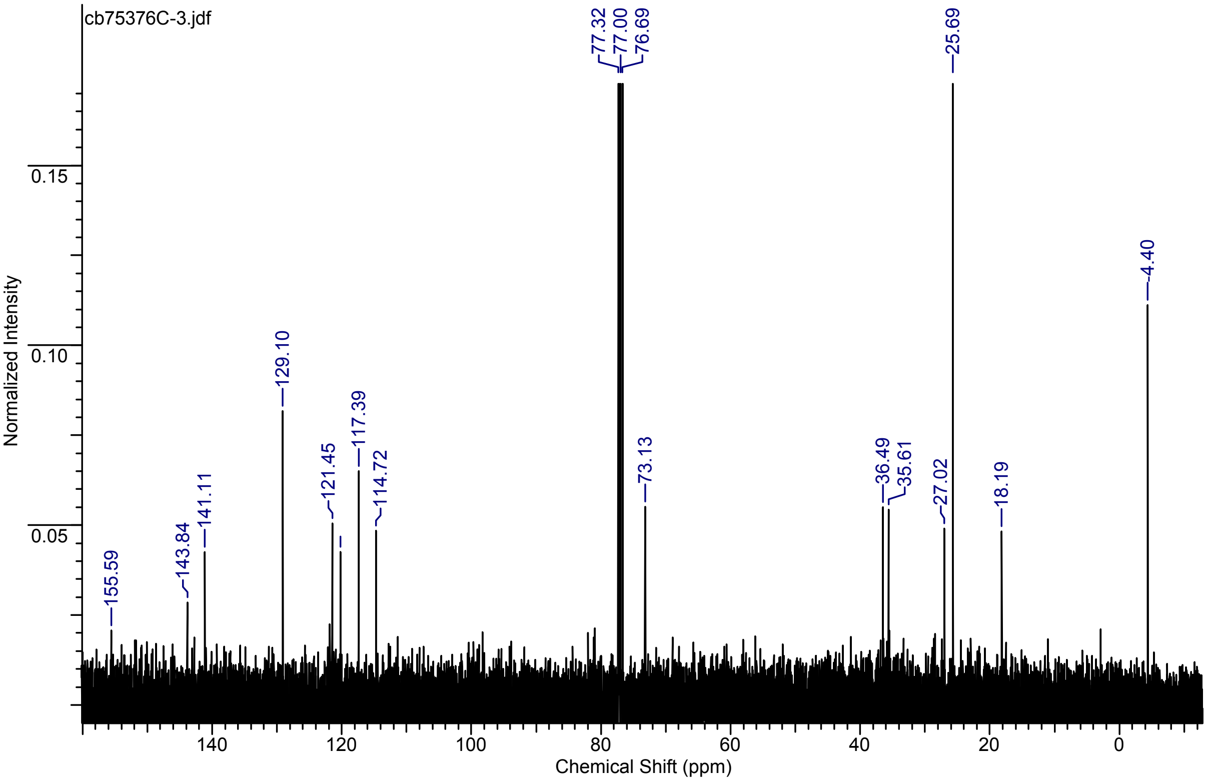
cb75376C-3.jdf
Normalized Intensity
Chemical Shift (ppm)
155.59
143.84
141.11
129.10
121.45
117.39
114.72
77.32
77.00
76.69
73.13
36.49
35.61
27.02
25.69
18.19
-4.40

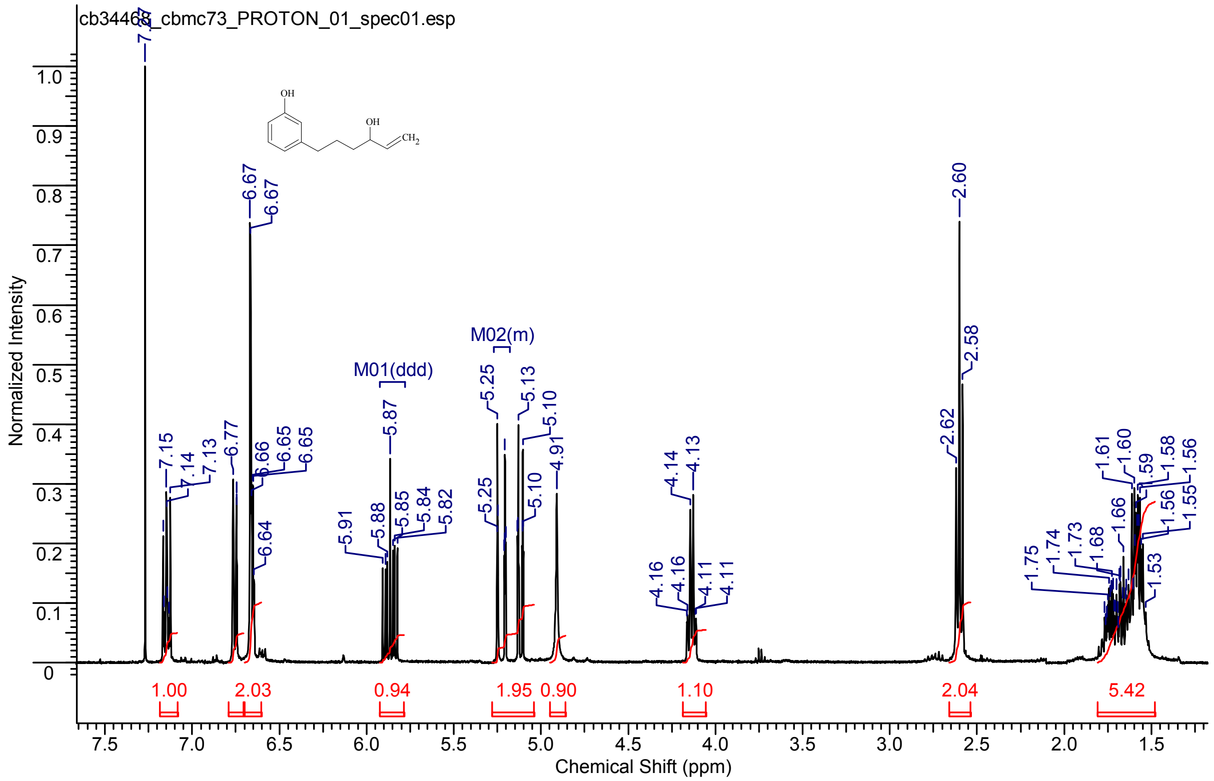

cb34468_cbmc73_PROTON_01_spec01.esp
OH
OH
CH2
M01(ddd)
M02(m)
7.27
7.15
7.14
7.13
6.77
6.67
6.67
6.66
6.65
6.65
6.64
5.91
5.88
5.87
5.85
5.84
5.82
5.25
5.25
5.13
5.10
5.10
4.91
4.16
4.16
4.14
4.13
4.11
4.11
2.62
2.60
2.58
1.75
1.74
1.73
1.68
1.66
1.61
1.60
1.59
1.58
1.56
1.56
1.55
1.53
1.00
2.03
0.94
1.95
0.90
1.10
2.04
5.42
Normalized Intensity
Chemical Shift (ppm)

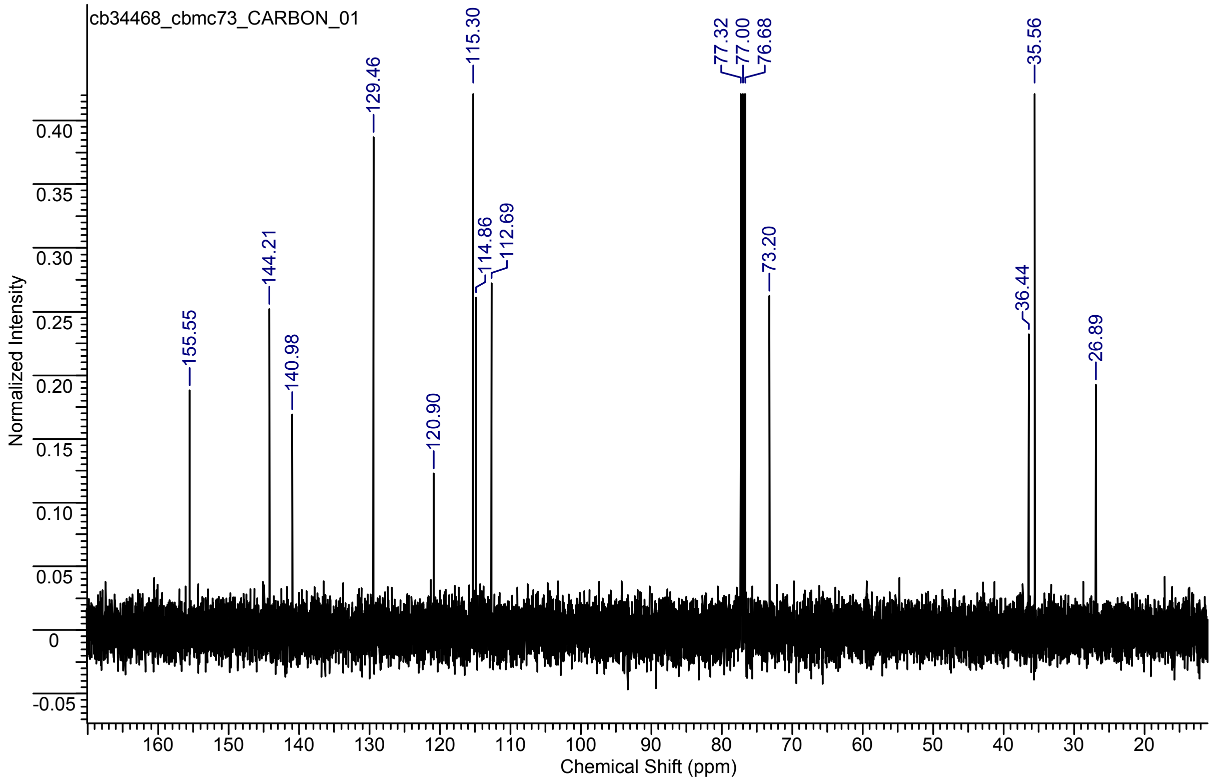

cb34468_cbmc73_CARBON_01
155.55
144.21
140.98
129.46
120.90
115.30
114.86
112.69
77.32
77.00
76.68
73.20
36.44
35.56
26.89
Normalized Intensity
Chemical Shift (ppm)

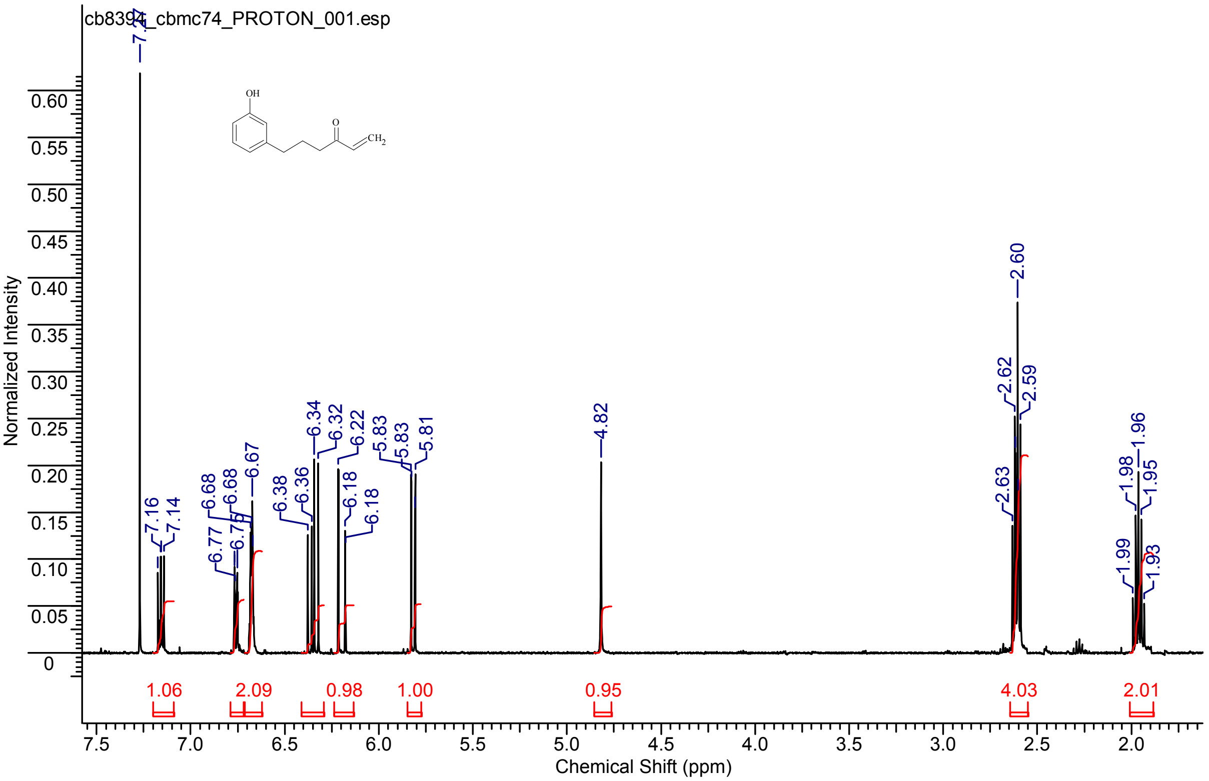
cb8394_cbmc74_PROTON_001.esp
OH
O
CH2
Normalized Intensity
Chemical Shift (ppm)
1.06
2.09
0.98
1.00
0.95
4.03
2.01

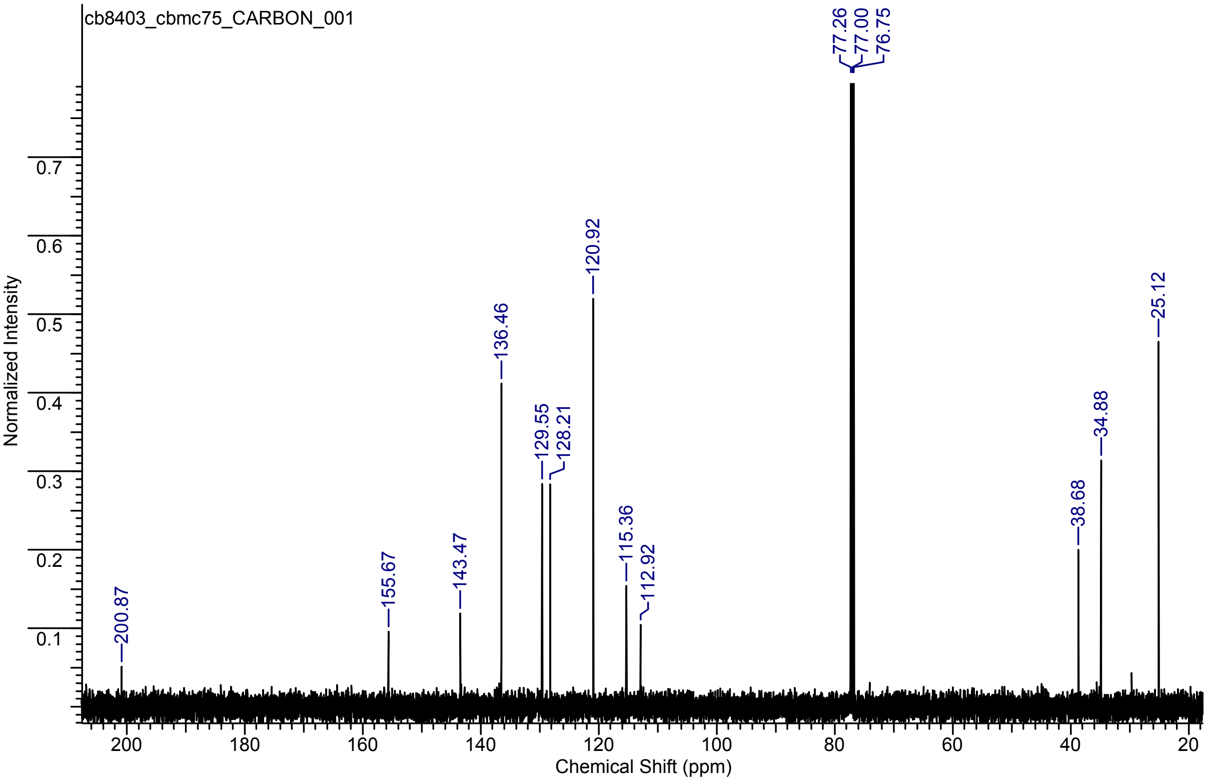
cb8403_cbmc75_CARBON_001
Normalized Intensity
Chemical Shift (ppm)
200.87
155.67
143.47
136.46
129.55
128.21
120.92
115.36
112.92
77.26
77.00
76.75
38.68
34.88
25.12

1. R. L. Winston and M. C. Fitzgerald, *Anal. Biochem.*, 1998, **262**, 83-85.
2. L. J. Gazzard, W. B. Motherwell and D. A. Sandham, *J. Chem. Soc., Perkin Trans. 1*, 1999, 979-993.
3. B. Schmidt, *J. Org. Chem.*, 2004, **69**, 7672-7687.
4. A. R. Katritzky, G. F. Zhang and J. L. Jiang, *J. Org. Chem.*, 1995, **60**, 7589-7596.
5. M. Pal and S. L. Bearne, *Org. & Biomol. Chem.*, 2014, **12**, 9760-9763.
6. D. R. Morton and J. L. Thompson, *J. Org. Chem.*, 1978, **43**, 2102-2106.
7. U. E. Hille, Q. Hu, C. Vock, M. Negri, M. Bartels, U. Mueller-Vieira, T. Lauterbach and R. W. Hartmann, *Eur. J. Med. Chem.*, 2009, **44**, 2765-2775.
8. X.-C. Cai, X. Wu and B. B. Snider, *Org. Lett.*, 2010, **12**, 1600-1603.
9. H. Stetter and A. Mertens, *Chem. Ber. Recl.*, 1981, **114**, 2479-2490.
10. R. D. Peterson, C. A. Theimer, H. Wu and J. Feigon, *J. Biomol. NMR*, 2004, **28**, 59-67.
11. F. Delaglio, S. Grzesiek, G. W. Vuister, G. Zhu, J. Pfeifer and A. Bax, *J. Biomol. NMR.*, 1995, **6**, 277-293.
12. R. Fogh, J. Ionides, E. Ulrich, W. Boucher, W. Vranken, J. P. Linge, M. Habeck, W. Rieping, T. N. Bhat, J. Westbrook, K. Henrick, G. Gilliland, H. Berman, J. Thornton, M. Nilges, J. Markley and E. Laue, *Nat. Struct. Biol.*, 2002, **9**, 416-418.
13. W. F. Vranken, W. Boucher, T. J. Stevens, R. H. Fogh, A. Pajon, P. Llinas, E. L. Ulrich, J. L. Markley, J. Ionides and E. D. Laue, *Proteins: Struct. Funct. Bioinform.*, 2005, **59**, 687-696.
14. M. Pellecchia, P. Sebbel, U. Hermanns, K. Wuthrich and R. Glockshuber, *Nat. Struct. Biol.*, 1999, **6**, 336-339.
15. W. F. Vranken, W. Boucher, T. J. Stevens, R. H. Fogh, A. Pajon, M. Llinas, E. L. Ulrich, J. L. Markley, J. Ionides and E. D. Laue, *Proteins*, 2005, **59**, 687-696.
16. M. Fossi, H. Oschkinat, M. Nilges and L. J. Ball, *J Magn Reson*, 2005, **175**, 92-102.
17. A. J. Nederveen, J. F. Doreleijers, W. Vranken, Z. Miller, C. A. Spronk, S. B. Nabuurs, P. Guntert, M. Livny, J. L. Markley, M. Nilges, E. L. Ulrich, R. Kaptein and A. M. Bonvin, *Proteins*, 2005, **59**, 662-672.
18. W. L. DeLano, DeLano Scientific, San Carlos, CA, USA, 2002.
19. J. Liang, H. Edelsbrunner and C. Woodward, *Protein Sci.*, 1998, **7**, 1884-1897.
